# Controlling the transmembrane transport of chloride by dynamic covalent chemistry with azines†

**DOI:** 10.1039/d4sc08580a

**Published:** 2025-01-27

**Authors:** Marcin Konopka, Lau Halgreen, Anca-Elena Dascalu, Matúš Chvojka, Hennie Valkenier

**Affiliations:** a Engineering of Molecular NanoSystems, Université libre de Bruxelles (ULB) Avenue F.D. Roosevelt 50, CP165/64 B-1050 Brussels Belgium hennie.valkenier@ulb.be; b Department of Chemistry and RECETOX Faculty of Science, Masaryk University Brno 62500 Czech Republic

## Abstract

Stimuli-responsive transmembrane ion transport has become a prominent area of research due to its fundamental importance in cellular processes and potential therapeutic applications. Commonly used stimuli include pH, light, and reduction or oxidation agents. This paper presents the use of dynamic covalent chemistry to activate and modulate the transmembrane transport of chloride in liposomes. An active chloride transporter was obtained *in situ* within the lipid bilayer by dynamic azine metathesis. The transport activity was further tuned by changing the structure of the added azines, while the dynamic covalent chemistry could be activated by lowering the pH. This dynamic covalent chemistry opens a new approach towards controlling transmembrane transport.

## Abstract 2

This article presents the use of dynamic covalent chemistry in liposomal membranes to activate and modulate the transmembrane transport of chloride by addition of different azine-containing compounds.

## Graphical abstract

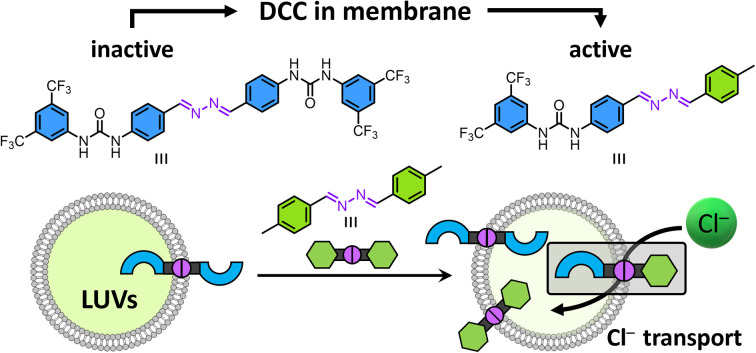

## Introduction

In recent years, the development of synthetic ion transporters has become an area of great interest, due to the key role of transmembrane transport in metabolic processes in living organisms.^1–3^ Various diseases are linked to deficient or poorly regulated anion transport and synthetic ion transporters hold therapeutic potential for such channelopathies.^4,5^ Additionally, synthetic anion transporters have also been employed to disrupt homeostasis of cancer or bacterial cells.^6–8^ For both types of applications, precise control over the activation of the anion transport is desirable. Examples of stimuli-responsive transport systems have been reported, starting with pH-controlled anion transporters,^9,10^ and followed by numerous transporters that are controlled or activated by light and redox reactions and that have been discussed in three recent reviews.^11–13^

Dynamic Covalent Chemistry (DCvC) is a well-established concept for the synthesis and *in situ* modification of organic structures and supramolecular systems by covalent bonds which become reversible under specific conditions. Examples of such reversible bonds include imines, acyl-hydrazones, disulfides, boronic acids, *ortho*-esters, and tetrazines.^14–17^ DCvC has already been used in various applications, such as drug delivery,^18^ gene transfection,^19^ thiol-mediated uptake,^20^ dynamic self-assembly of amphiphiles,^21–23^ or in other smart biomaterials.^24,25^

However, applications of DCvC in well-defined molecular structures for ion transport applications are rare. An imine-based system has been employed to transport Ca^2+^ cations through a chloroform phase in U-tube experiments.^26,27^ Ion channels have been formed by the DCvC of acylhydrazones to obtain helical structures^28^ or by linking two cyclodextrin units *via* disulfides inside the membrane.^29^ Bravin and Hunter have shown that the lipid membrane of artificial liposomes can be used as a template, amplifying the concentration of lipophilic product in a dynamic combinatorial library based on thiols and Michael receptors.^30^ In a follow-up study, the same DCvC was used to transport thiols into liposomes for the activation of a caged enzyme encapsulated inside vesicles, demonstrating transmembrane signal transduction.^31^ Finally, imine formation was used to enhance the transport of amino acids by synthetic anions transporters.^32^

Although the cited examples have shown some applications of DCvC in the context of lipid membranes or ion transport, to the best of our knowledge, controlling the transmembrane transport of ions by employing dynamic covalent chemistry (DCvC) within lipid bilayers has not yet been demonstrated. One of the challenges to be overcome has been the need for a dynamic covalent functional group that is sufficiently stable in the lipid/aqueous environment, while at the same time achieving an exchange equilibrium within the timescale on which the stability of the liposomes can be ensured. While imines showed very rapid hydrolysis in membrane environments and the exchange rate of acylhydrazones was too slow for our purposes, we recently discovered that azines can be used as a functional group in DCvC.^33^ Azines showed good stability in the presence of water and fast exchange (<1 h) in the presence of acid as catalyst. Here we show that DCvC of azines can be used within lipid bilayers to activate and tune the transmembrane transport of chloride by synthetic receptors.

## Results and discussion

### Design and synthesis of transporters

Our first aim was to develop a system where the target transporter is dynamically synthesised *in situ* in the lipid membrane from building blocks *via* azine metathesis. Azine metathesis is a reaction similar to imine metathesis in which the substituents of different azines are exchanged, resulting in a dynamic mixture of asymmetric and symmetric products at thermodynamic equilibrium.^33^
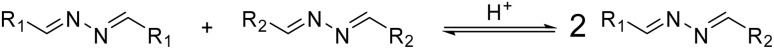


**Fig. 1:**
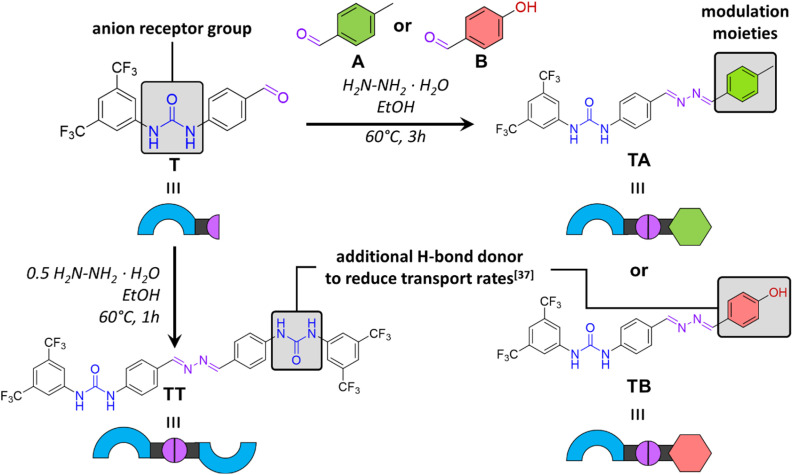
Design and synthesis of chloride transporters containing reversible azine bonds.

To control anion transport *via* azine metathesis, it was crucial to design both active and inactive transporters with an azine group. Therefore, we started with building block T, consisting of a benzaldehyde with a bis(trifluoromethyl)-phenylurea as the anion receptor unit (Fig. 1). The latter is a well-studied motif for the efficient binding and transport of anions such as Cl^−^.^34–36^ The aldehyde function can be readily transformed into an azine upon reaction with hydrazine and another aldehyde, which serves as modulator for the transport activity.

We recently reported that H-bond donor groups, which are not involved in the binding of the anion, strongly inhibit the anion transport activity.^37^ For instance, while *o*-phenylenebisureas are highly active anion transporters,^38^ the corresponding *m*-phenylenebisureas showed hardly any transport activity.^37^ This was attributed to the interaction of the second anion binding site with the phospholipid headgroups, acting as an anchor and preventing transport. Based on this finding, we hypothesised that symmetric azine TT would be a poor anion transporter. On the other hand, combining T with the simple 4-methylbenzaldehyde A would yield azine TA, that we expected to be active as an anion transporter. We also tested 4-hydroxybenzaldehyde B as modulator, predicting that the resulting compound TB, with an additional H-bond donor, would likely exhibit poorer transport activity due to increased interactions with the lipid headgroups and water.

Building block T was prepared in two steps, starting with the reaction of 4-aminobenzyl alcohol with 3,5-bis(trifluoromethyl)phenyl isocyanate to form a urea, followed by the oxidation of the alcohol group into an aldehyde using MnO_2_. The reaction of aldehyde T with 0.5 equiv. of hydrazine hydrate in ethanol with trifluoroacetic acid resulted in symmetrical azine TT in 83% yield. The synthesis of asymmetric azines TA and TB was performed following a similar protocol, using 1 equiv. of each aldehyde and 1 equiv. of hydrazine hydrate, to provide a mixture of compounds from which the desired asymmetric azines TA and TB could be isolated by column chromatography in 35% and 27% yield, respectively. Detailed synthetic procedures and characterisation data are provided in the ESI.†

### Anion transport tests of the new compounds

The next step was to verify our hypothesis regarding the anion transport activity of the synthesised compounds T, TT, TA, and TB. For this, we have chosen the commonly used lucigenin assay because it is a convenient method that can be used at different pH levels.^39^ As azine metathesis was found to be most efficient in the presence of an acid^33^ and we wanted to monitor the transmembrane transport of Cl^−^ during the DCvC experiments, we have chosen to work at pH 5. The fluorescent probe lucigenin was thus encapsulated in liposomes composed of POPC and cholesterol (7 : 3 ratio) in a solution of 225 mM NaNO_3_ and 5 mM MES at pH 5. A pulse of NaCl was added to the exterior of the liposomes to create a Cl^−^ gradient of 25 mM and the transport process was monitored by fluorescence spectroscopy. When Cl^−^ is transported into the liposomes, it quenches the fluorescence of lucigenin. The NaNO_3_ solution is used to allow the displacement of charge upon transport of Cl^−^ into the liposomes to be balanced by the transport of NO_3_^−^ out of the liposomes *via* an antiport mechanism.

**Fig. 2:**
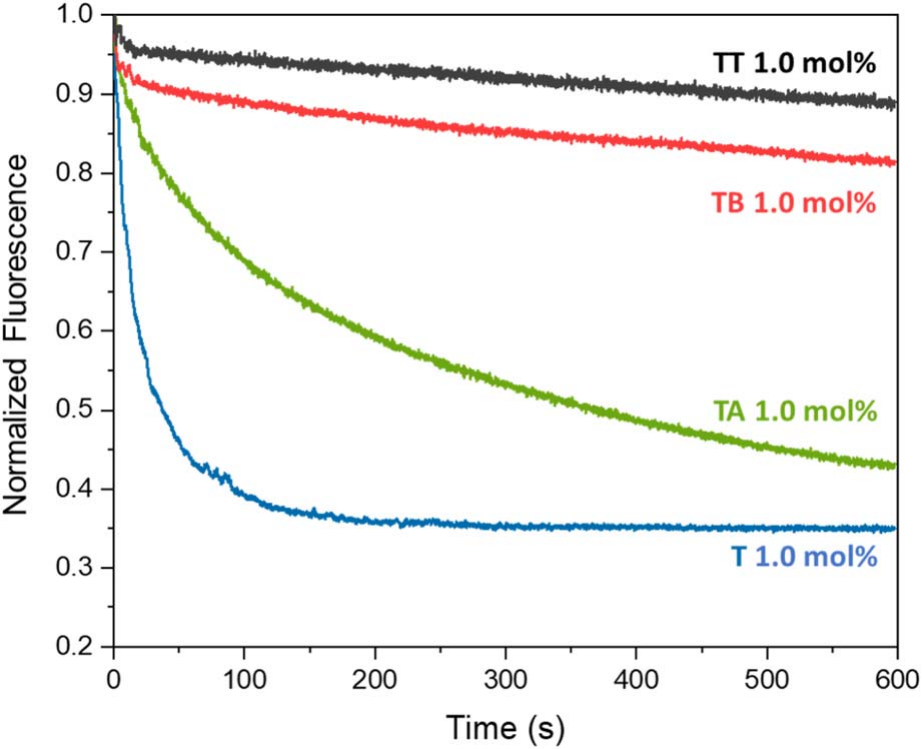
Average transport curves for compounds T, TT, TA and TB (1 mol%) upon addition of NaCl (25 mM) in the lucigenin assay (0.4 mM of lipids POPC/cholesterol in 7 : 3 ratio, 0.8 mM lucigenin inside the liposomes, in 225 mM NaNO_3_, 5 mM MES buffer, pH 5).

The different compounds were added to the liposomes at a concentration of 1 mol% with respect to the lipid concentration (0.4 mM) and the transport results are shown in Fig. 2. As assumed, aldehyde T is a good anion transporter, similar to its analogue without the aldehyde group.^37^ We did not observe any pH dependence of the anion transport by T when comparing its activity at pH 5 and pH 7 (Fig. S16†).

Azine TT was poorly soluble in methanol or acetonitrile and had to be preincorporated into the membrane of the liposomes during their formation. In agreement with our hypothesis that the two different urea binding sites would inhibit its transport, TT showed no significant anion transport activity. ^1^H NMR experiments showed that TT has no significant solubility in water, but that this compound can indeed be incorporated into the lipid bilayer of liposomes (Fig. S28†), indicating that the absence of transport by TT is not caused by the absence of this compound from the membrane. Azine TT is thus present in the membrane, but inactive as an anion transporter. This was also the case for smaller azine TB, which has an OH group that can interact with the phospholipid headgroups. Encouragingly, the more lipophilic azine TA did show clear anion transport activity. Higher rates of transport by TA were observed upon increasing its concentration (Fig. S17†).

### Azine metathesis

**Fig. 3:**
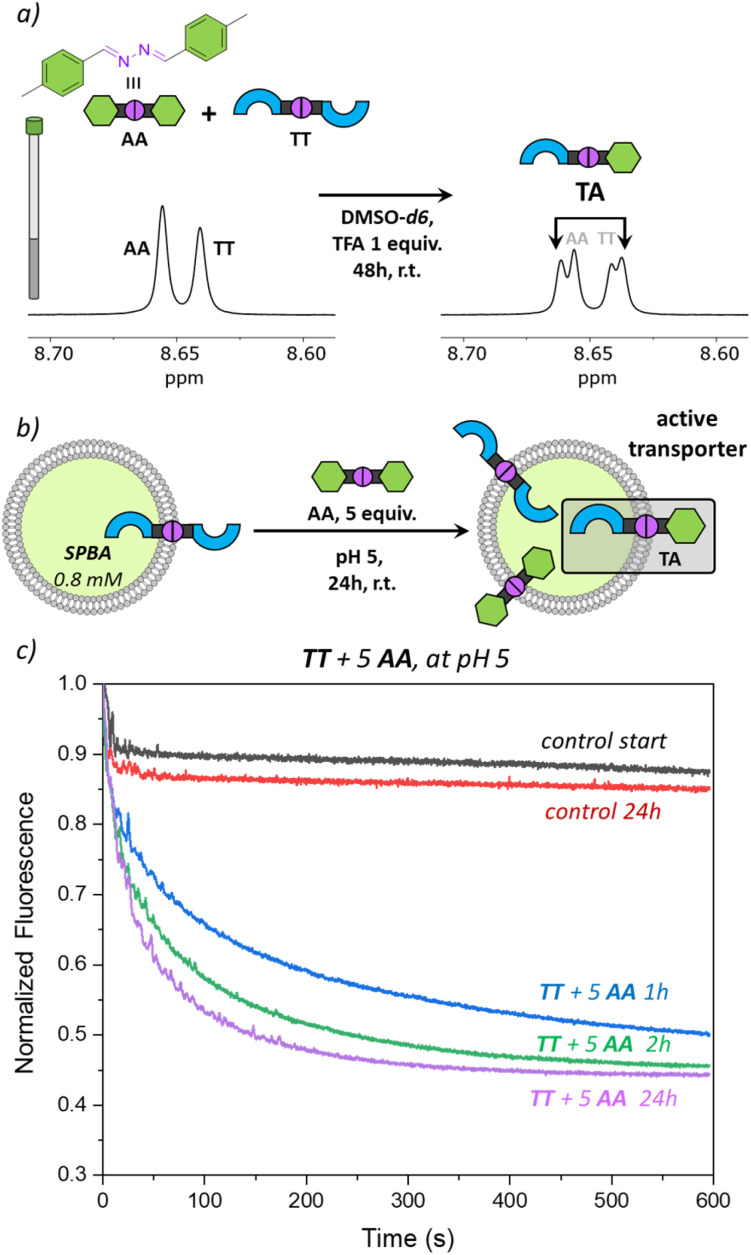
(a) Scheme of the DCvC reaction between AA and TT in DMSO-*d*_6_ and ^1^H NMR spectra prior to TFA addition (left) and 48 h after TFA addition (right), (b) scheme of the DCvC reaction performed inside the liposomal membrane, starting with preincorporated TT (1 mol%) to which AA (5 mol%) was added; (c) transport curves as monitored by the fluorescence of SPBA (0.8 mM) upon addition of NaCl (25 mM) to liposomes (0.4 mM of lipids POPC/cholesterol in 7 : 3 ratio, in 225 mM NaNO_3_, 5 mM MES at pH 5).

To validate the potential of TA as an active transporter, we first ensured that the azine metathesis reaction between TT and AA could proceed effectively. To demonstrate this, we combined equimolar solutions of TT and AA (5 mM) in DMSO-*d*_6_ in NMR tubes. One tube served as a control, while 1 equiv. of TFA was added to the second tube. After 48 h, we recorded the ^1^H NMR spectra of both samples. The acidified sample revealed a new set of signals, demonstrating the successful azine metathesis and the formation of the asymmetric azine TA (Fig. 3a). The presence of the TT, AA, and TA in this sample was confirmed by mass spectrometry (Fig. S15†). The control tube showed no changes after 48 h, confirming that acidic conditions are essential for the azine metathesis reaction.

### Activating transport with dynamic covalent chemistry

Having found that TT is not active as a transporter, while TA is able to perform anion transport and that azine metathesis between TT and AA can give TA, we set out to perform this reaction *in situ* in the liposomal membrane (Fig. 3b). Not knowing the timescale required for the azine metathesis reaction inside the membrane, we replaced the fluorescent probe lucigenin (*N*,*N*′-dimethyl-9,9′-biacridinium dinitrate) by its more hydrophilic analogue SPBA (*N*,*N*′-bis(3-sulfonatopropyl)-9,9′-biacridinium), which is less prone to leak out of the liposomes over time.^39^ Before starting the DCvC experiments, we first needed to ensure that the azine TT is stable in the membrane and does not hydrolyse over time to release aldehyde T, as this is an active anion transporter. We have preincorporated TT at 1 mol% in liposomes with SPBA encapsulated and for the rest identical conditions to those described above. Transport was tested directly after preparation of the liposomes with TT (black curve in Fig. 3c) and again after 24 h (red curve). No significant transport was observed for both cases, indicating that there is no significant hydrolysis of TT taking place in the lipid membranes, highlighting again the remarkable stability of azines compared to imines.

This control experiment paved the way for DCvC experiments, which were performed in glass vials containing 3 mL of liposome solution (0.4 mM lipids, 12 nmol of TT, 1 equiv.) at room temperature with continuous stirring. The symmetrical azine AA (12 μL, 5.0 mM in MeCN, 5 equiv.) was added to the liposomes and transport runs were recorded at different intervals after the addition of azine AA. A very clear transport activity was observed already after 1 hour (Fig. 3c). This shows that azine AA was effectively delivered into the membrane where the azine metathesis between preincorporated TT and AA occurred, resulting in the formation of the active anion transporter TA. Control experiments in which AA was added to liposomes without TT did not show any transport (Fig. S21†). The transport activity in the DCvC transport experiments increased from 1 h to 2 h, while hardly any further improvement was observed after 24 h, indicating that the thermodynamic equilibrium of the exchange reaction is reached after ∼2 h. A concentration of TA of ∼1.7 mol% is then expected statistically (see Table S1†).

### Transport experiments with dynamic combinatorial libraries of azines

**Fig. 4:**
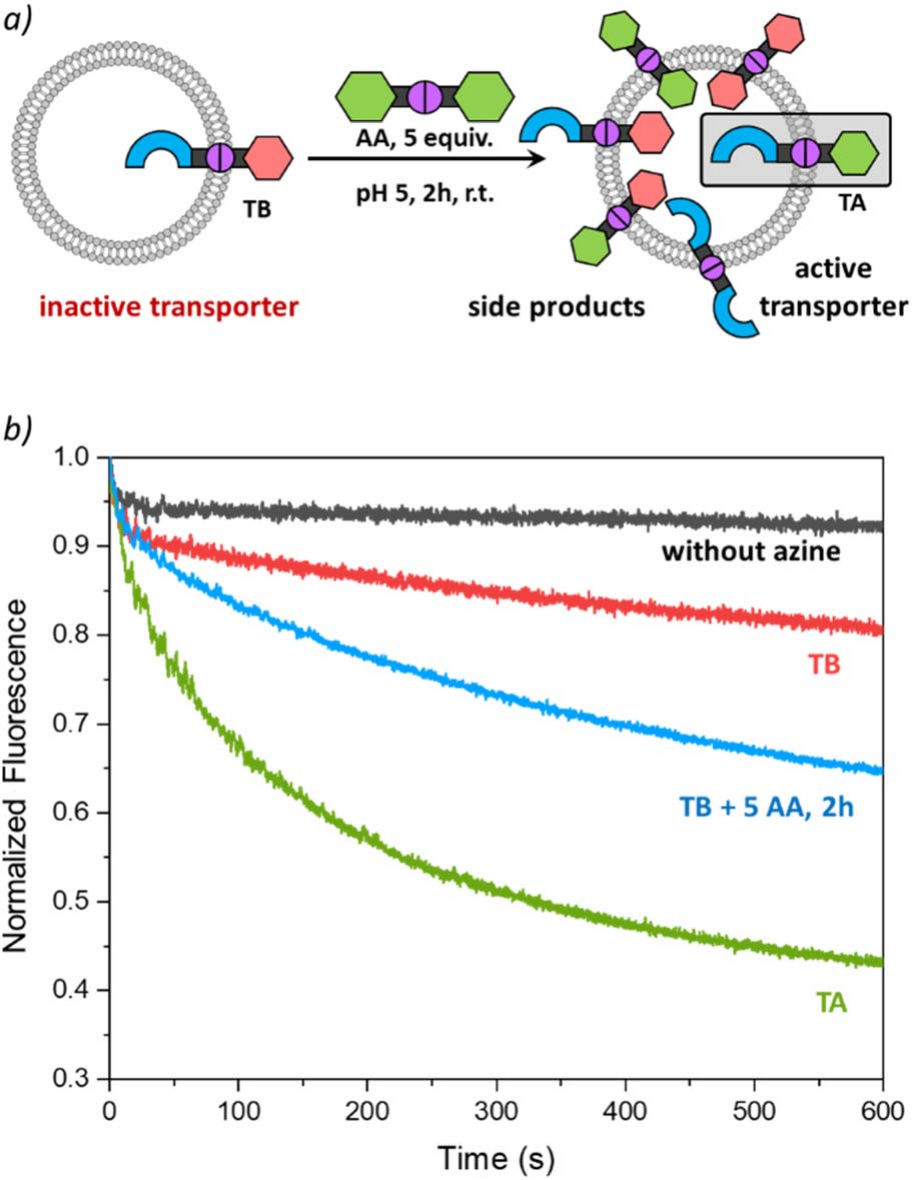
(a) Scheme of the formation of a dynamic combinatorial library inside the liposomal membrane; (b) transport curve recorded 2 h after the addition of the azines TB (1 mol%) + AA (5 mol%) to the liposomes by monitoring the fluorescence of lucigenin (0.8 mM) upon addition of NaCl (25 mM) to liposomes (0.4 mM of lipids POPC/cholesterol in 7 : 3 ratio, in 225 mM NaNO_3_, 5 mM MES at pH 5). The curves obtained without any azines added or upon addition of only TB or TA (1 mol%) are shown for comparison.

Dynamic combinatorial libraries are obtained when multiple building blocks for DCvC are combined to form more complex mixtures of products. The experiment described above contains only three products from two different aldehyde building blocks. We were keen to test if we could increase the complexity of the mixture in the membrane to three building blocks and thus six potential azines (Fig. 4a).

In this experiment we started with lucigenin containing liposomes without any azine and a control run was recorded. Then 1 mol% of TB was post-inserted by adding a solution of TB in acetonitrile to the liposomes and, after 3 minutes, a transport measurement was recorded, showing very little transport activity (Fig. 4b, red curve). Next, 5 equiv. of AA were added to the liposomes already containing TB and, after 2 h, that sample showed clear transport of Cl^−^ (Fig. 4b, blue curve). During the DCvC inside the liposomal membranes, TB and AA could react to form a mixture of TA, TB, TT, AA, AB, BB, of which only TA is an active anion transporter, expected to be formed at maximum 0.8 mol%. The observation of clear transport upon mixing TB and AA thus indicates that TA has been formed in this more complex dynamic combinatorial library inside the membrane.

### Modulation of transport rates with different azines

**Fig. 5:**
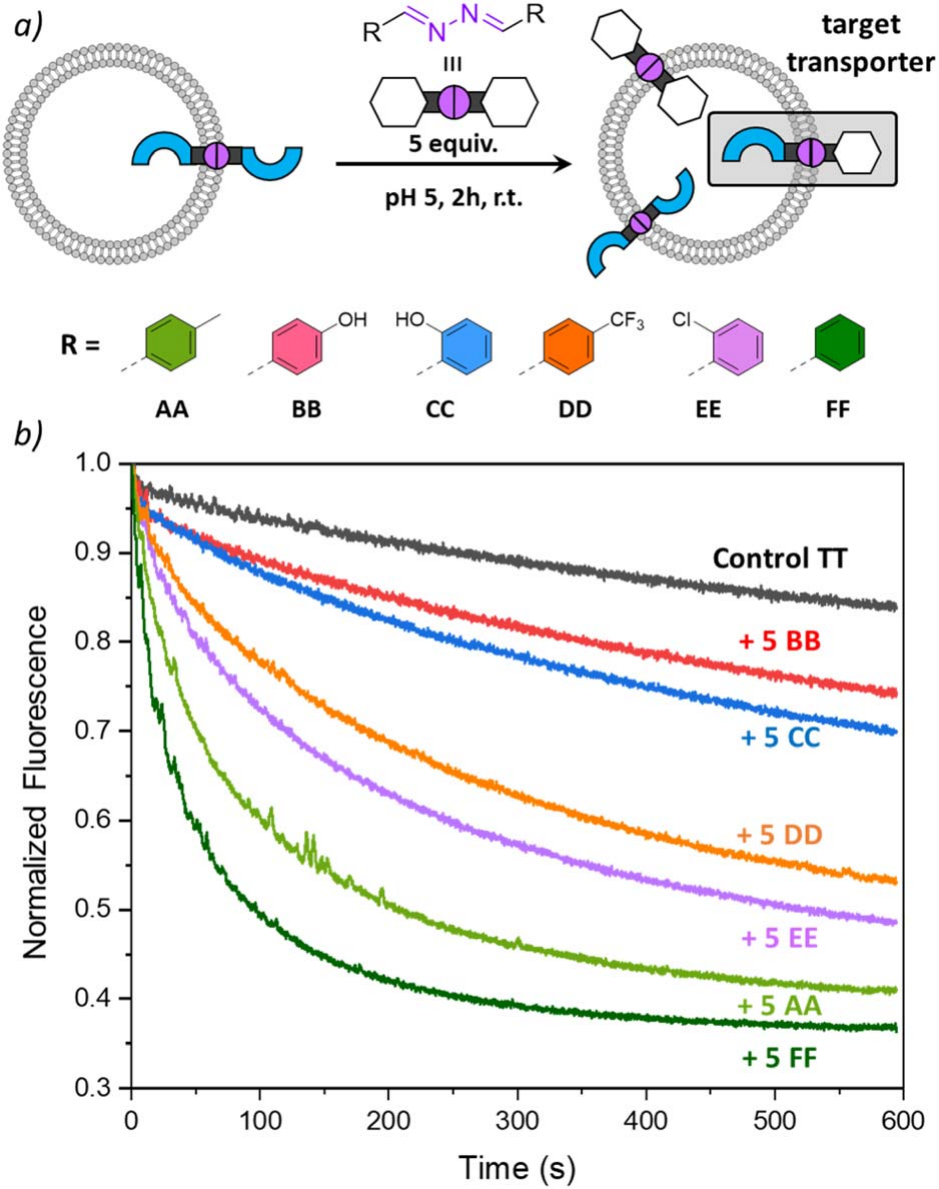
(a) Scheme of the DCvC reactions performed inside the liposomal membrane, starting with preincorporated TT (1 mol%) to which AA–FF (5 mol%) were added; (b) transport curves recorded 2 h after addition of the azines AA–FF by monitoring the fluorescence of lucigenin (0.8 mM) upon addition of NaCl (25 mM) to liposomes (0.4 mM of lipids POPC/cholesterol in 7 : 3 ratio, in 225 mM NaNO_3_, 5 mM MES at pH 5).

In the experiments so far, we have shown that DCvC with azines made of transporter building block T and modulators A and B could take place inside liposomal membranes to induce anion transport. To investigate whether other modulator building blocks could be used in a similar way, we tested azines CC–FF, obtained from benzaldehyde and its derivatives (Fig. 5 and ESI page 2†).^33^ Building block C resembles building block B in that it also contains a hydroxyl H-bond donor group, while D–F are less polar building blocks. Azine TT was again preincorporated at 1 mol% in liposomes with lucigenin encapsulated and different azines AA–FF were added to the liposomes. After 2 h, anion transport runs were recorded, and the results are shown in Fig. 5.

The highest transport rates were observed for samples to which apolar azines AA and FF had been added, to form active azines TA and TF, respectively. The addition of halogenated azines DD and EE resulted in slightly lower transport rates, but still formed active transporters TD and TE. The hydroxyl-containing azines BB and CC did not give rise to any significant improvement of anion transport. This could be due to the membrane anchoring of TB and TC, but, considering the low lipophilicity of azines BB and CC (cLogP 2.9), it cannot be excluded that they partition less efficiently into the liposomal membranes upon their addition and that less TB or TC is formed. Experiments in which the transport was recorded 1 h after the addition of AA–FF showed similar results and are shown in Fig. S24.† Together with the results in Fig. 5, these clearly show that the anion transport activity can be tuned by DCvC upon addition of different azines.

### Activating transport with pH

**Fig. 6:**
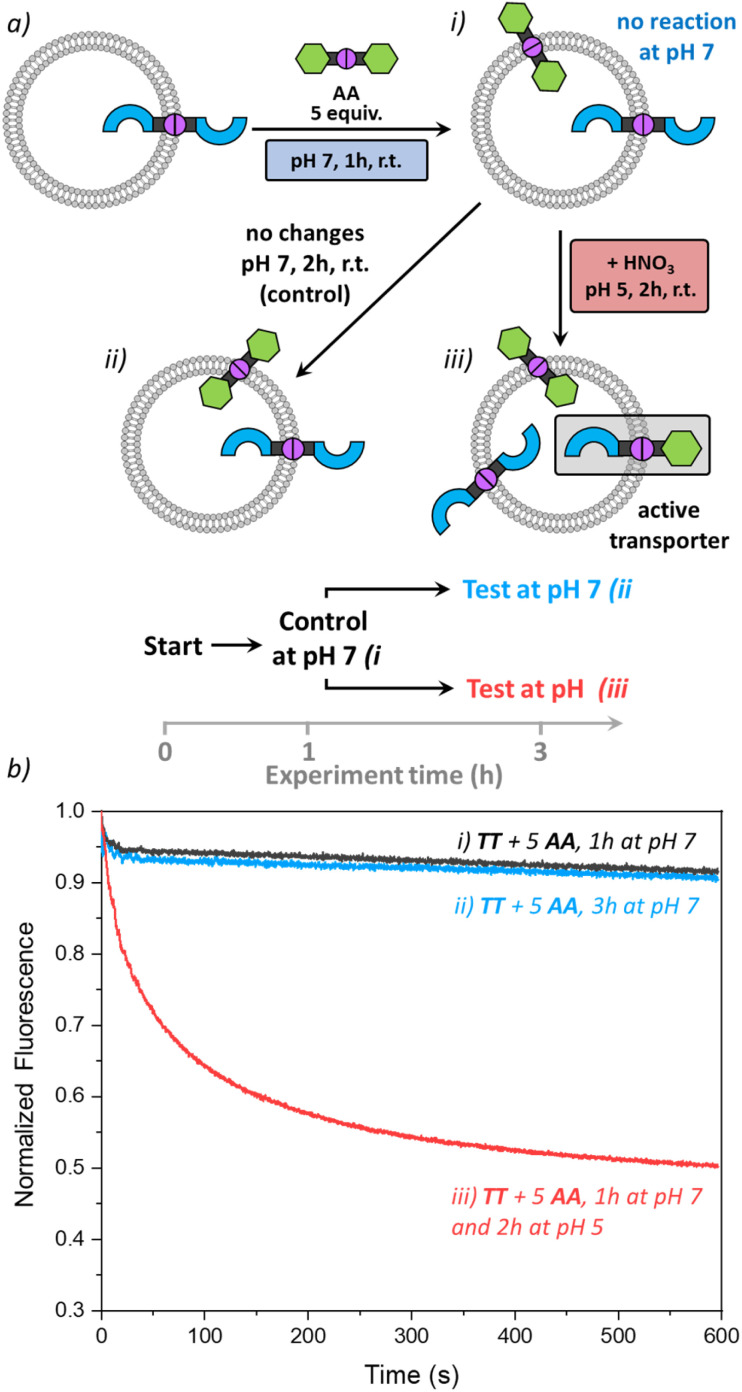
(a) Scheme of the pH activation experiments performed, starting with preincorporated TT (1 mol%) and addition of AA (5 mol%) at pH 7 (225 mM NaNO_3_, 5 mM HEPES at pH 7, i), after which part of the liposomes were kept at pH 7 (ii) and the other part was acidified to pH 5 with HNO_3_ (iii); (b) transport curves recorded 1 h after addition of AA (i), or an additional 2 hours after that (ii and iii) by monitoring the fluorescence of lucigenin (0.8 mM) upon addition of NaCl (25 mM) to liposomes (0.4 mM of lipids POPC/cholesterol in 7 : 3 ratio).

As the DCvC of azines was reported to require acid as a catalyst,^33^ we wondered if we could utilise pH as an additional stimulus to activate anion transport. For this study, we used liposomes with preincorporated TT (1 mol%), but now at pH 7 (225 mM NaNO_3_, 5 mM HEPES). Then, 5 mol% AA was added, and no transport was observed after 1 h (Fig. 6, i). The remaining liposome suspension was divided into two parts. The first part remained as a control sample at pH 7. The second part was acidified by addition of dilute nitric acid to reach pH 5.

Then the anion transport in both samples was tested after 2 h. The acidified sample showed clear transport of Cl^−^ (Fig. 6, iii), comparable to previous results obtained in liposomes originally prepared at pH 5 (Fig. 3). The control sample remained unchanged with no transport observed (Fig. 6, ii). Indeed, even after 24 h no active TA was formed at pH 7 (see Fig. S26†). These results show that we can control the azine metathesis inside the membrane externally by changing the pH of the environment, which is relevant in biological contexts.

### The impact of membrane fluidity on transport and dynamic covalent chemistry

In addition to the pH, the membrane fluidity may influence the DCvC reaction as well. To study this, we prepared LUVs using dipalmitoylphosphatidylcholine (DPPC), which undergoes a gel-to-liquid phase transition at 41 °C, with compound TT preincorporated (1 mol%) at AA added (5 mol%) at pH 5. Equilibration and transport were tested at 25 °C and 45 °C (see Section 8 of the ESI†). At 25 °C, when DPPC is in the solid ordered phase, no transport was observed for any of the samples, confirming that these compounds act as mobile carriers. When the samples were incubated for 1–2 h at 45 °C followed by a transport experiment at this temperature, very fast transport was observed. Interestingly, incubation of the samples at 25 °C for either 1 or 2 h followed by transport experiments at 45 °C gave identical transport curves, but with significantly lower rates of transport than when the sample was incubated at 45 °C. This indicates that no significant DCvC takes place in DPPC at 25 °C, while at this temperature almost full equilibration is obtained in POPC. The membrane fluidity is thus a crucial factor for both the DCvC and the transport.

## Conclusions

In this study, we have demonstrated a new approach to the external control of transmembrane Cl^−^ transport using dynamic covalent chemistry with azines. Firstly, we have successfully demonstrated that dynamic azine metathesis can be performed inside lipid membranes. This chemistry was used to induce transmembrane Cl^−^ transport upon the addition of azines as an external stimulus. An active Cl^−^ transporter was synthesised *in situ* inside the liposomal membrane from an inactive pre-transporter. We demonstrated that it is possible to use more complex mixtures of building blocks or to tune the rate of anion transport by the structure of the azine added. We also showed that pH can be used as an additional stimulus to activate the dynamic covalent chemistry in the membrane. We have thus shown that, in addition to pH, redox and light, dynamic covalent chemistry can be used as a strategy to control transmembrane transport processes. While the potential toxicity of azines and hydrazines might prevent therapeutical applications of the current compounds, we hope that our proof of concept will inspire future research into the control of transmembrane transport by dynamic covalent chemistry.

## Data availability

The datasets supporting this article have been uploaded as part of the ESI.†

## Author contributions

Conceptualisation and methodology: MK, HV. Investigation, synthesis and characterisation: MK, LH, AED, MC; transport studies: MK. Visualisation and validation: MK, HV. Writing original draft: MK. Writing, review and editing: MK, HV, LH, AED, MC. Funding acquisition: MK, HV. Project administration and supervision: HV. All authors reviewed the results and approved the final version of the manuscript.

## Conflicts of interest

The authors declare no conflicts of interest.

## Supplementary Material

SC-016-D4SC08580A-s001
