# Supplementary material for: Controlling the transmembrane transport of chloride by dynamic covalent chemistry with azines
Source: Chem Sci. 2025 Jan 27;16(8):3509–15. doi: 10.1039/d4sc08580a (PMC11770589; doi:10.1039/d4sc08580a)
Supplement: SC-016-D4SC08580A-s001 [file SC-016-D4SC08580A-s001.pdf]

Supplementary Information (SI) for Chemical Science.
This journal is © The Royal Society of Chemistry 2025

Electronic Supporting Information for

Controlling the Transmembrane Transport of Chloride by Dynamic Covalent Chemistry with Azines

Marcin Konopka,[a] Lau Halgreen,[a] Anca-Elena Dascalu,[a] Matúš Chvojka,[a,b] Hennie Valkenier [a]*

[a] *Université libre de Bruxelles (ULB), Engineering of Molecular NanoSystems, Avenue F.D. Roosevelt 50, CP165/64, B-1050 Brussels, Belgium.*
[b] *Department of Chemistry and RECETOX Faculty of Science, Masaryk University, Brno 62500, Czech Republic.*
* hennie.valkenier@ulb.be

Table of Contents

1. Materials and methods 2
2. Compounds list 2
3. Synthesis 3
   Synthesis of **T-OH** 3
   Synthesis of aldehyde **T** 3
   Synthesis of azine **TT** 4
   Synthesis of azine **TA** 4
   Synthesis of azine **TB** 4
   Synthesis of azine **BB** 5
   Synthesis of azine **CC** 5
4. NMR Spectra 6
5. NMR experiments on azine metathesis 12
6. Transport studies with the lucigenin assay 14
   Liposome preparation 14
   Anion transport measurements 14
   Results of the anion transport measurements 15
7. Dynamic products distribution 21
8. Membrane fluidity impact on azines metathesis 22
9. Study of the incorporation of TT in the liposomal membrane 23

1. Materials and methods

All commercially available reagents were purchased from Sigma Aldrich, Merck, or Alfa Aesar and used without further purification. Deuterated solvents were used for as such without being dried.

^{1}H and ^{13}C{^{1}H} NMR spectra were recorded on a Jeol JNM-ECZ400R/S3 spectrometer equipped with a 5 mm NM Royal probe at 298 K. The ^{1}H residual signals of the deuterated solvent were used as a reference.

HRMS spectra were measured on an Agilent QTOF 6520 or 6546 by electron spray ionization (ESI-MS).

All transport studies were recorded on a Horiba Fluoromax 4 fluorescence spectrometer.

2. Compounds list

Abbrev.	Structure	cLogP		Abbrev.	Structure	cLogP
**T**	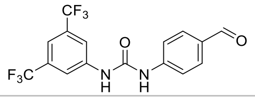	5.0		**AB**	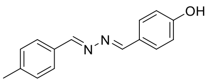	4.0
**TT**	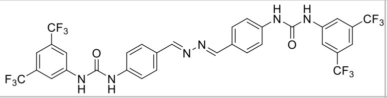	10.9		**T-OH**	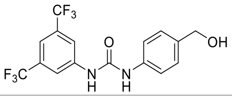	4.5
**TA**	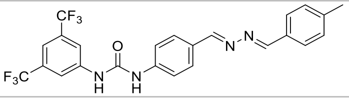	8.1		**AA**	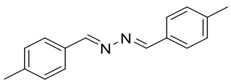	5.2
**TB**	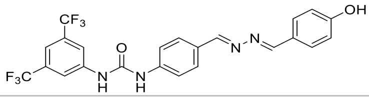	6.9		**BB**	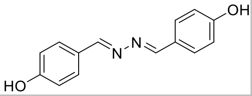	2.9
**TC**	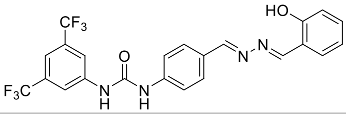	6.9		**CC**	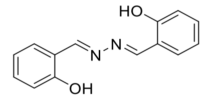	2.9
**TD**	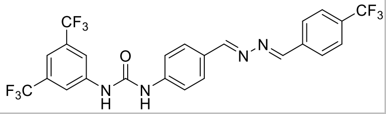	8.4		**DD**	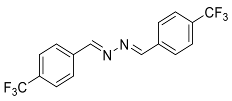	6.0
**TE**	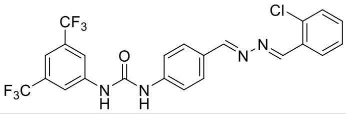	8.3		**EE**	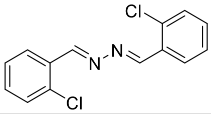	5.6
**TF**	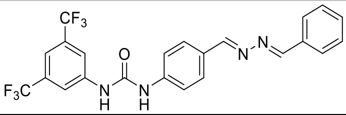	7.6		**FF**	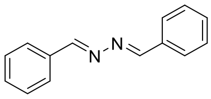	4.2

3. Synthesis

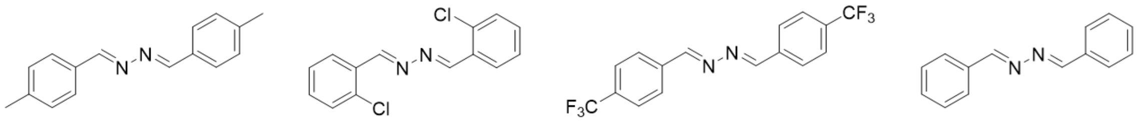

AA **DD** **EE** **FF**

Azines **AA, DD, EE** and **FF** were synthesised in our laboratory and reported previously.[1]

Synthesis of **T-OH**

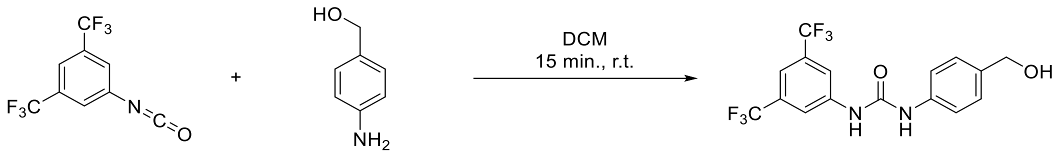

The solutions of 3,5-bis(trifluoromethyl)phenylisocyanate (500 mg, 2.0 mmol, 1 equiv.) and 4-aminobenzyl alcohol (240 mg, 2.0 mmol, 1 equiv.) in 5 mL DCM were combined in the 50 mL round-bottom flask. Immediately after mixing a white solid crashed out from the mixture. After an additional 15 minutes of stirring, the white solids were filtered off, washed with 20 mL DCM and dried under a high vacuum. Yield: 570 mg (76%).

^{1}H NMR (400 MHz, DMSO-d_6) δ 9.35 (s, 1H), 8.92 (s, 1H), 8.12 (s, 2H), 7.62 (s, 1H), 7.43 (d, *J* = 8.5 Hz, 2H), 7.24 (d, *J* = 8.6 Hz, 2H), 5.08 (t, *J* = 5.7 Hz, 1H), 4.44 (d, *J* = 5.5 Hz, 2H). **^{13}C NMR** (100 MHz, DMSO-d_6) δ 152.44, 141.93, 137.56, 136.73, 130.69 (q, $J_{C\text{-}F}$ = 32.4 Hz), 127.10, 123.33 (q, $J_{C\text{-}F}$ = 273.5 Hz), 118.66, 117.90, 114.26, 62.61. **HRMS** (ESI+) m/z calc. for $C_{16}H_{13}F_6N_2O_2$ [M+H$^+$] 379.0876, found 379.0890.

Synthesis of aldehyde **T**

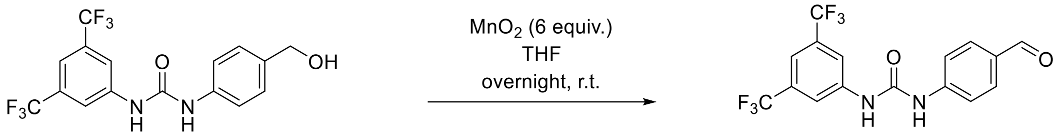

Solid MnO_2 (522 mg, 6.0 mmol, 6 equiv.) was added to the solution of **T-OH** (378 mg, 1.0 mmol, 1 equiv.) in 10 mL of THF in r.t. This mixture was sonicated for 2 minutes to activate the MnO_2 and then stirred overnight in r.t. After that the mixture was filtered through the celite pad, which was then washed with an additional portion of THF (50 mL). The collected filtrate was evaporated in *vacuo* resulting in a pale-yellow solid. The crude product was suspended in 10 mL of DCM and sonicated for 2 minutes. After that the white solids were filtered off, washed with DCM (50 mL) and dried under a high vacuum. Yield: 380 mg (73%).

^{1}H NMR (400 MHz, DMSO-d_6) δ 9.87 (s, 1H), 9.51 (s, 2H), 8.19 – 8.09 (m, 2H), 7.91 – 7.80 (m, 2H), 7.73 – 7.68 (m, 2H), 7.67 (s, 1H). **^{13}C NMR** (100 MHz, DMSO-d_6) δ 191.41, 152.13, 144.96, 141.42, 130.92, 130.63, 130.76 (q, $J_{C\text{-}F}$ = 34.4 Hz), 123.28 (q, $J_{C\text{-}F}$ = 272.6 Hz), 118.35, 118.20, 114.87. **HRMS** (ESI+) m/z calc. for $C_{16}H_{11}F_6N_2O_2$ [M+H$^+$] 377.0719, found 377.0722.

---

[1] A.-E. Dascalu, L. Halgreen, A. Torres-Huerta and H. Valkenier, Dynamic covalent chemistry with azines, *Chem. Commun.*, 2022, **58**, 11103-11106.

Synthesis of azine **TT**

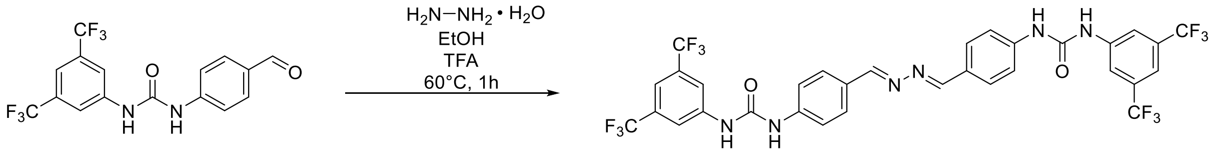

Aldehyde **T** (376 mg, 1.0 mmol, 1 equiv.) was dissolved in 10 mL EtOH. Then hydrazine hydrate (25 mg, 0.5 mmol, 0.5 equiv.) and TFA (57 mg, 0.5 mmol, 0.5 equiv.) were added under stirring. The mixture was heated at 60°C for 1 hour. After this time, a yellow solid precipitated from the solution. After cooling the mixture, the precipitate was filtered, rinsed with 10 mL of EtOH and dried under high vacuum. Yield: 310 mg (83%).

^{1}H NMR (400 MHz, DMSO-d_6) δ 9.47 (s, 2H), 9.31 (s, 2H), 8.64 (s, 2H), 8.15 (s, 4H), 7.82 (d, J = 8.9 Hz, 4H), 7.67 (s, 2H), 7.63 (d, J = 8.9 Hz, 4H). **^{13}C NMR** (100 MHz, DMSO-d_6) δ 160.64, 152.22, 141.91, 141.62, 130.74 (q, $J_{C\text{-}F}$ = 32.8 Hz), 129.24, 128.01, 123.31 (q, $J_{C\text{-}F}$ = 272.5 Hz), 118.57, 118.19, 114.66. **HRMS** (ESI+) m/z calc. for $C_{32}H_{21}F_{12}N_6O_2$ [M+H$^+$] 749.1529, found 749.1514.

Synthesis of azine **TA**

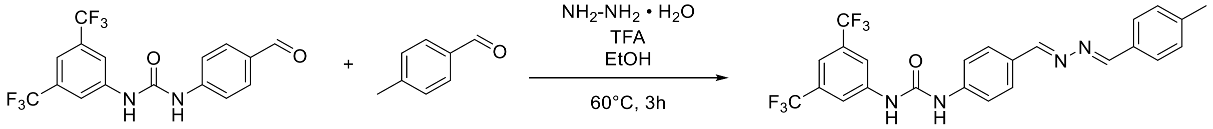

Aldehyde **T** (51 mg, 0.13 mmol), 4-methylbenzaldehyde (17 mg, 0.13 mmol) and hydrazine hydrate (6.8 µL, 0.13 mmol) were dissolved in 2 mL EtOH. Trifluoroacetic acid (15 mg, 0.13 mmol) was added and the reaction was stirred at 60°C for 3 hours. The resulting mixture of azines was evaporated onto celite and subjected to flash column chromatography using 50% ethyl acetate/heptane to yield the desired asymmetric azine as a pale-yellow solid. Yield: 24 mg (35%).

^{1}H NMR (400 MHz, DMSO-d_6) δ 9.48 (s, 1H), 9.32 (s, 1H), 8.66 (s, 1H), 8.64 (s, 1H), 8.15 (s, 2H), 7.82 (d, J = 8.9 Hz, 2H), 7.76 (d, J = 8.3 Hz, 2H), 7.67 (s, 1H), 7.63 (d, J = 8.9 Hz, 2H), 7.31 (d, J = 8.3 Hz, 2H), 2.37 (s, 3H). **^{13}C NMR** (100 MHz, DMSO-d_6) δ 160.96, 160.94, 152.23, 142.00, 141.62, 141.25, 131.30, 130.75 (q, $J_{C\text{-}F}$ = 33.1 Hz), 129.52, 129.31, 128.28, 127.93, 124.67, 123.31 (q, $J_{C\text{-}F}$ = 273.4 Hz), 118.58, 114.71, 21.16. **HRMS** (ESI+) m/z calc. for $C_{24}H_{18}F_6N_4O$ [M+H$^+$] 493.1458, found 493.1474.

Synthesis of azine **TB**

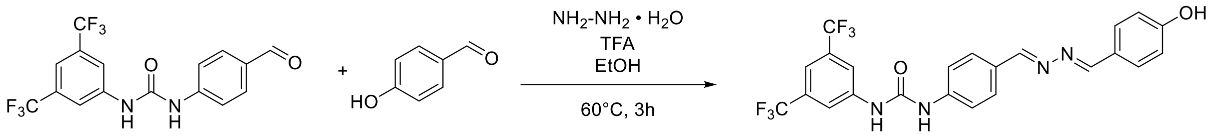

Aldehyde **T** (51 mg, 0.13 mmol), 4-hydroxybenzaldehyde (17 mg, 0.13 mmol) and hydrazine hydrate (6.8 µL, 0.13 mmol) were dissolved in 2 mL ethanol. Trifluoroacetic acid (15 mg, 0.13 mmol) was added and the reaction stirred at 60°C for 3 hours. The resulting mixture of azines was evaporated onto celite and subjected to flash column chromatography using 50% ethyl acetate/heptane followed by a second flash column using 25 % acetonitrile/toluene to yield the desired asymmetric azine as a pale-yellow solid. Yield: 18 mg (27%).

^{1}H NMR (400 MHz, DMSO-d_6) δ 10.09 (s, 1H), 9.47 (s, 1H), 9.30 (s, 1H), 8.60 (s, 1H), 8.59 (s, 1H), 8.15 (s, 2H), 7.80 (d, J = 9.0 Hz, 2H), 7.71 (d, J = 9.0 Hz, 2H), 7.67 (s, 1H), 7.62 (d, J = 8.9 Hz, 2H), 6.87 (d, J = 8.7 Hz, 2H). **^{13}C NMR** (100 MHz, DMSO-d_6) δ 160.97, 160.43, 160.06, 152.23, 141.76, 141.64, 130.5 (q, $J_{C\text{-}F}$ = 32.4 Hz), 130.24, 129.12, 128.15, 125.00, 123.32 (q, $J_{C\text{-}F}$ = 272.5 Hz), 118.58, 118.18, 115.78, 114.66. **HRMS** (ESI+) m/z calc. for $C_{23}H_{17}F_6N_4O_2$ [M+H$^+$] 495.1250, found 495.1268.

Synthesis of azine **BB**

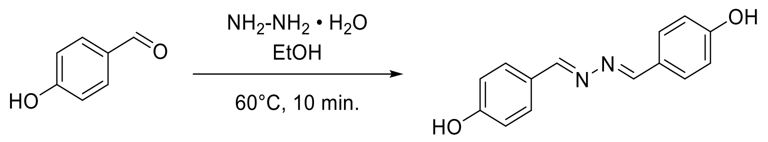

p-Hydroxybenzaldehyde (500 mg, 4.0 mmol, 2 equiv.) was dissolved in 5 mL absolute ethanol. Next, the hydrazine hydrate was added (102 µL, 2.0 mmol, 1 equiv.). The mixture was heated to 60°C for 10 min. and left to cool down. After 10 minutes pale yellow crystals crashed out. Crystals were filtered off, washed with 5 mL of EtOH and dried under a high vacuum. Yield: 425 mg (85%).

^{1}H NMR (400 MHz, DMSO-d_6) δ 10.06 (s, 2H), 8.55 (s, 2H), 7.69 (d, J = 8.7 Hz, 4H), 6.86 (d, J = 8.7 Hz, 4H). **^{13}C NMR** (100 MHz, DMSO-d_6) δ 160.37, 160.28, 130.09, 125.12, 115.75. **HRMS** (ESI+) m/z calc. for $C_{14}H_{13}N_2O_2$ [M+H]$^+$ 241.0972, found 241.1163.

Synthesis of azine **CC**

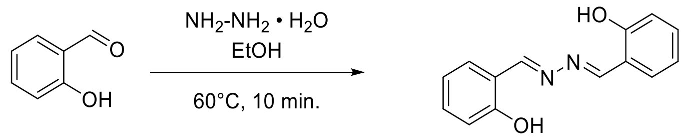

Salicylaldehyde (447 µL, 4.0 mmol, 2 equiv.) was dissolved in 5 mL absolute ethanol. Next, the hydrazine hydrate was added (102 µL, 2.0 mmol, 1 equiv.). The mixture was heated to 60°C for 10 min. and left to cool down. After 10 minutes pale yellow crystals crashed out. Crystals were filtered off, washed with 5 mL of EtOH and dried under a high vacuum. Yield: 440 mg (90%).

^{1}H NMR (400 MHz, $CDCl_3$) δ 11.39 (s, 2H), 8.72 (s, 2H), 7.45 – 7.33 (m, 4H), 7.04 (dd, J = 8.3, 1.1 Hz, 2H), 6.97 (td, J = 7.6, 7.5, 1.1 Hz, 2H). **^{13}C NMR** (100 MHz, $CDCl_3$) δ 164.70, 159.80, 133.44, 132.55, 119.72, 117.27, 117.15. In agreement with previously reported data.[2]

[2] D.-X. Xie, Z.-J. Ran, Z. Jin, X.-B. Zhang and D.-L. An, A simple fluorescent probe for Zn(II) based on the aggregation-induced emission, *Dyes and Pigments*, 2013, **96**, 495-499.

4. NMR Spectra

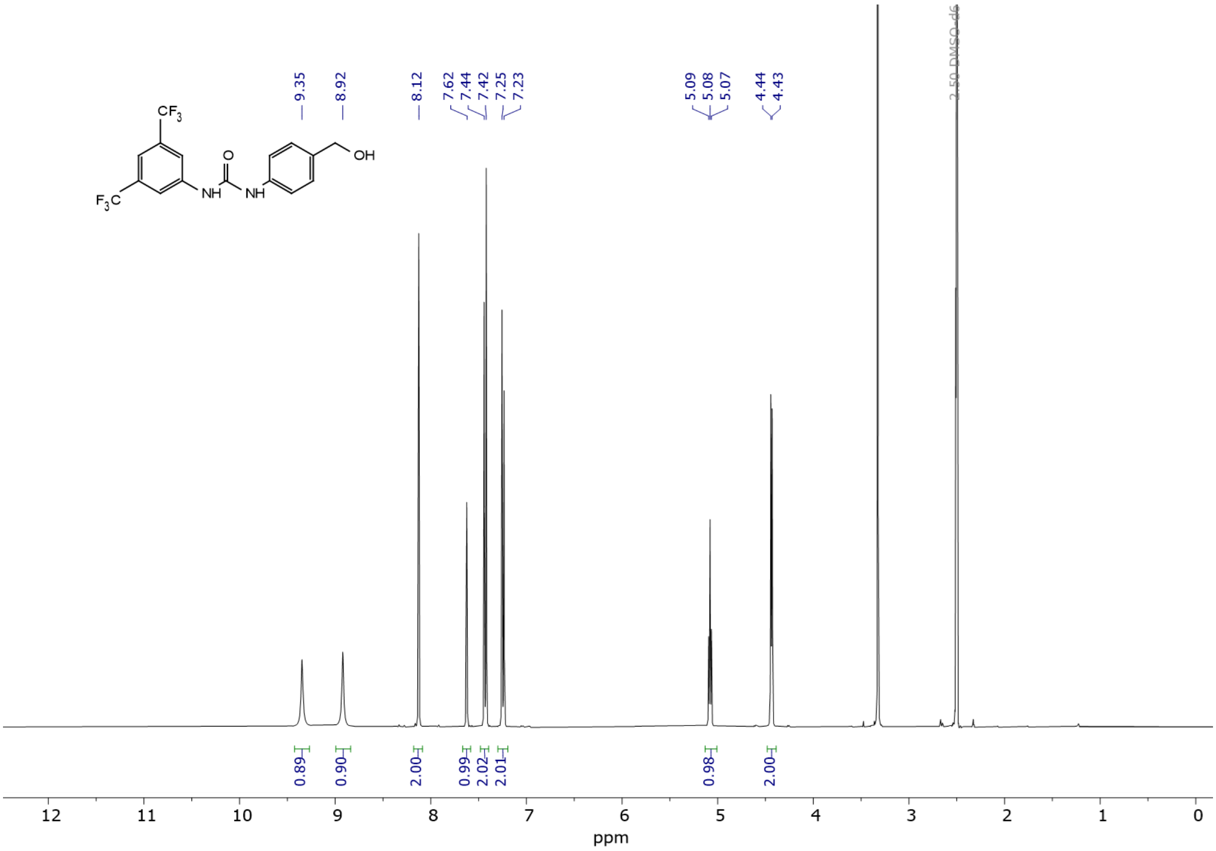

Figure S1. ^{1}H NMR spectrum of **T-OH** (DMSO-d$_6$, 400 MHz, 298 K).

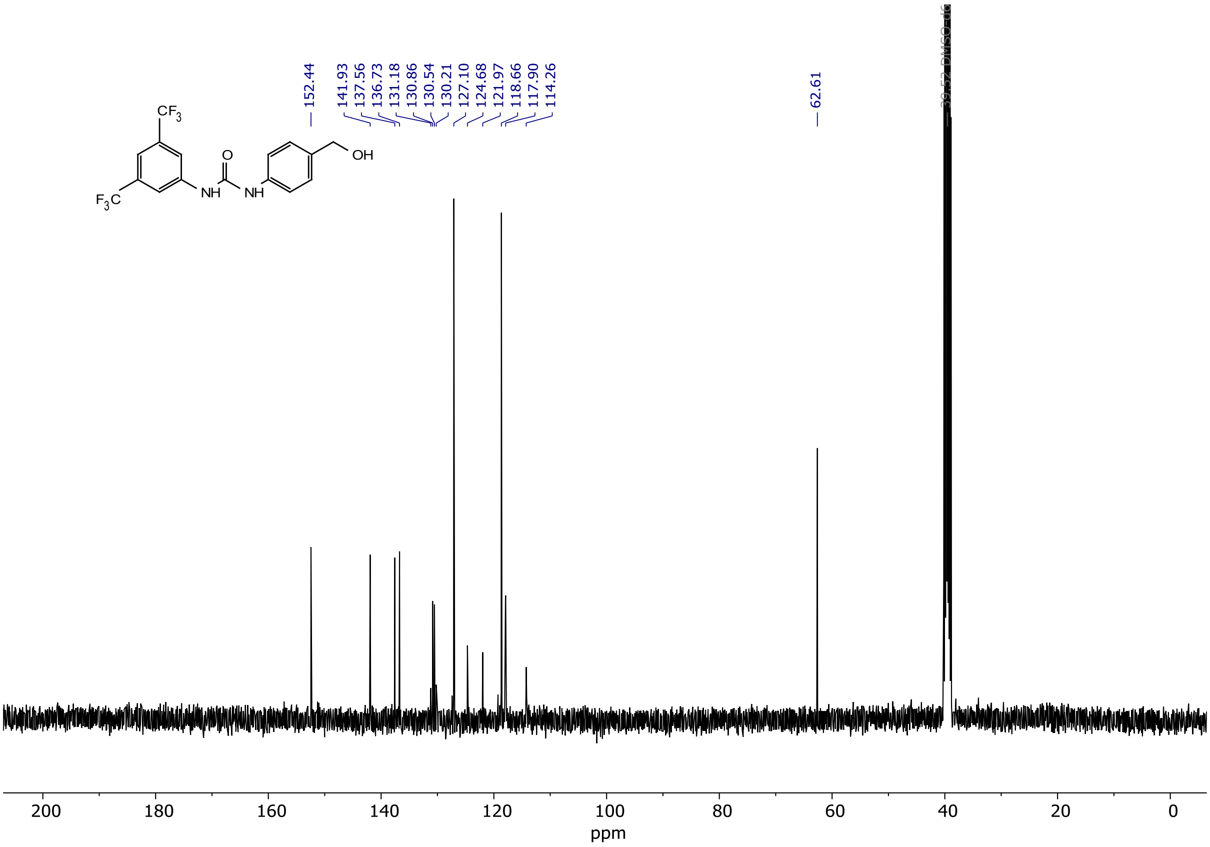

Figure S2. $^{13}C\{^{1}H\}$ NMR spectrum of **T-OH** (DMSO-d$_6$, 100 MHz, 298 K).

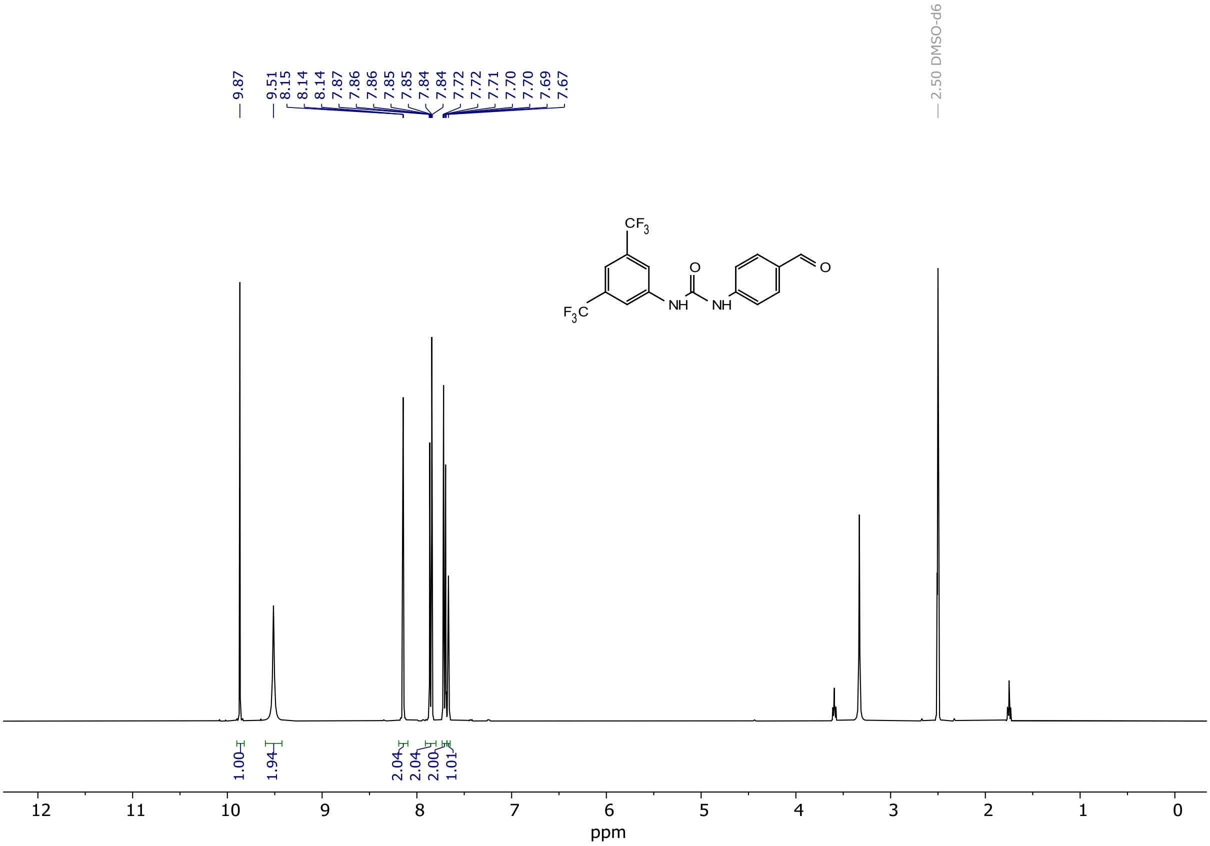

Figure S3. ^{1}H NMR spectrum of **T** (DMSO-d$_6$, 400 MHz, 298 K).

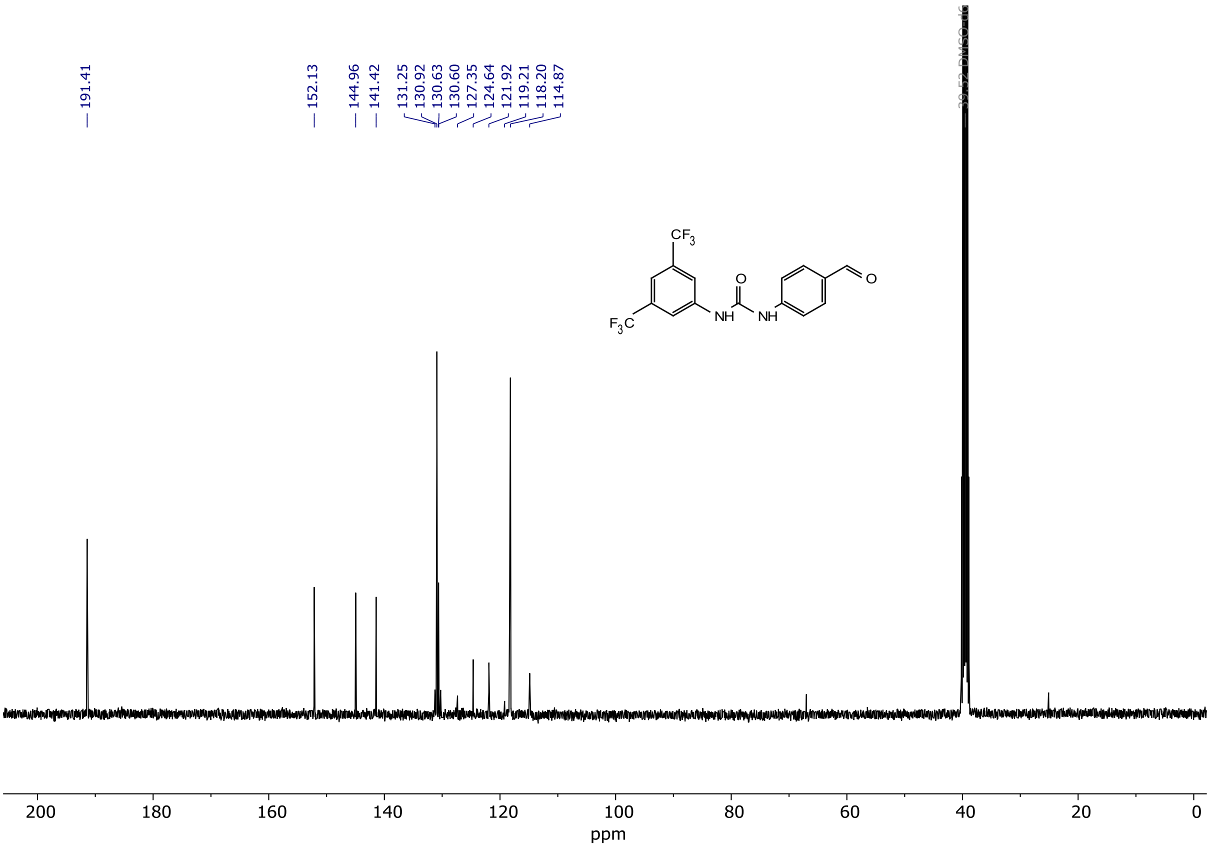

Figure S4. $^{13}C\{^{1}H\}$ NMR spectrum of **T** (DMSO-d$_6$, 100 MHz, 298 K).

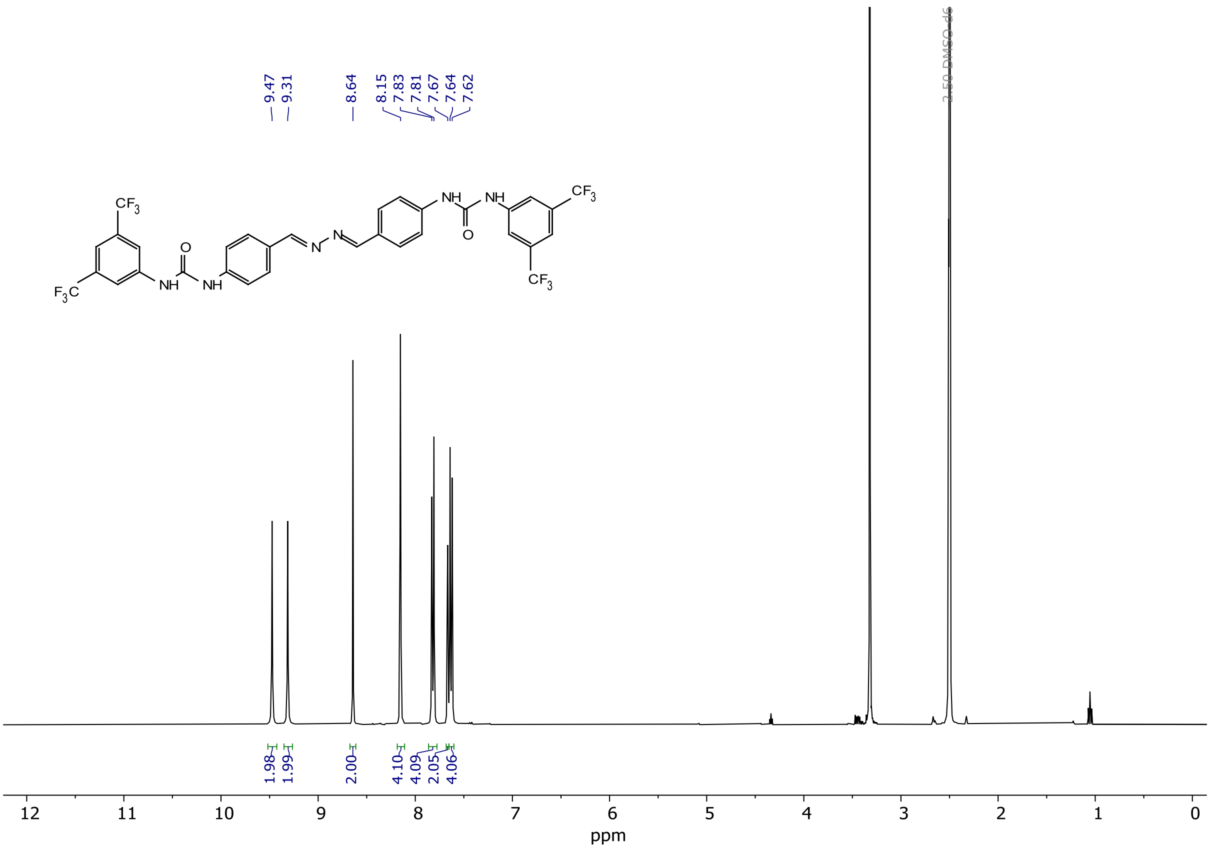

Figure S5. ^{1}H NMR spectrum of **TT** (DMSO-d_6, 400 MHz, 298 K).

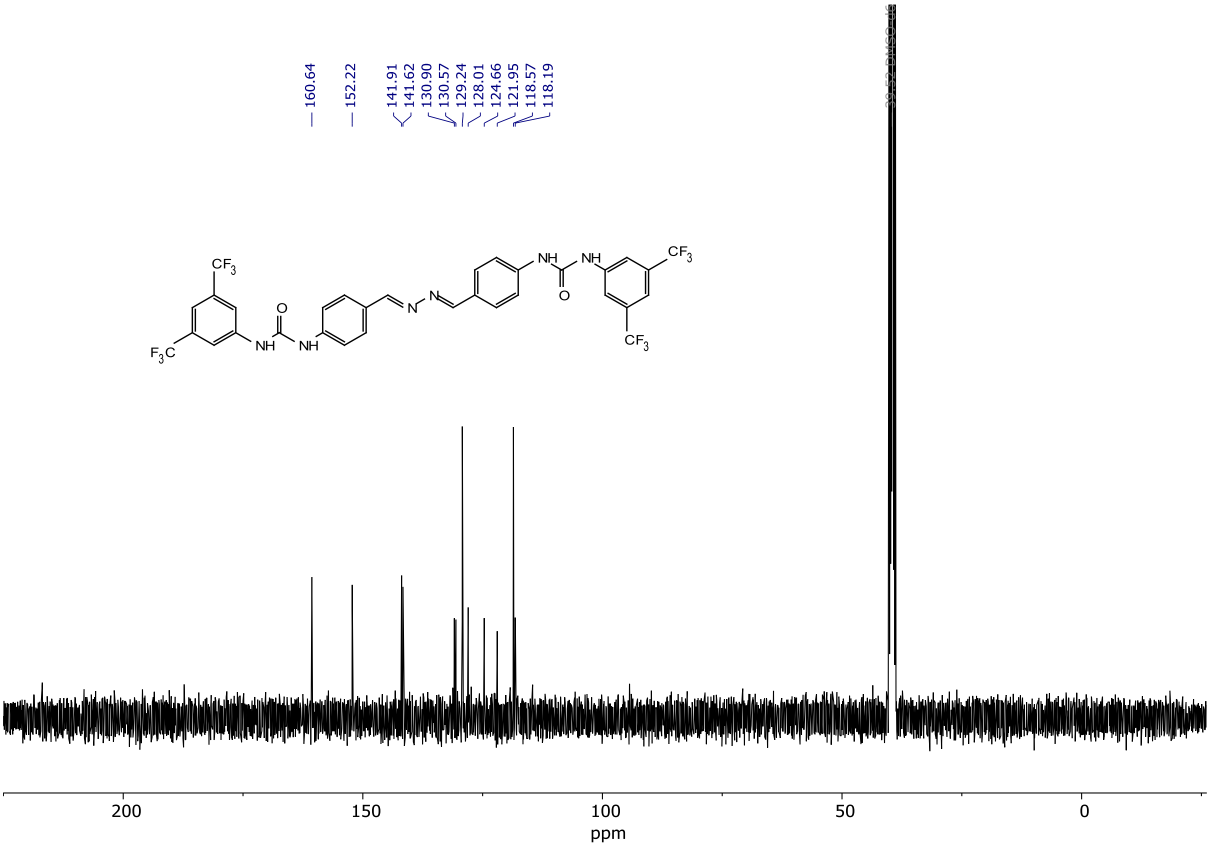

Figure S6. ^{13}C{^{1}H} NMR spectrum of **TT** (DMSO-d_6, 100 MHz, 298 K).

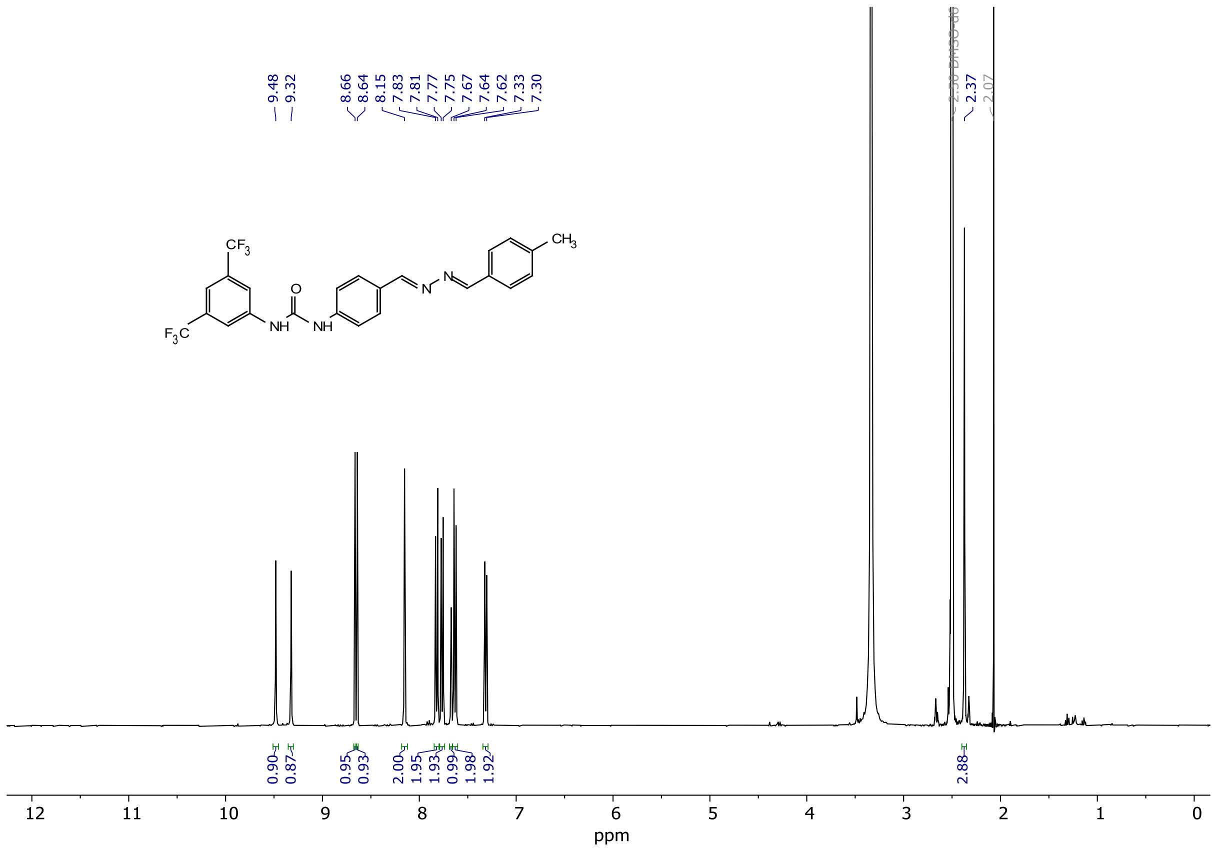

Figure S7. ^{1}H NMR spectrum of **TA** (DMSO-d_6, 400 MHz, 298 K).

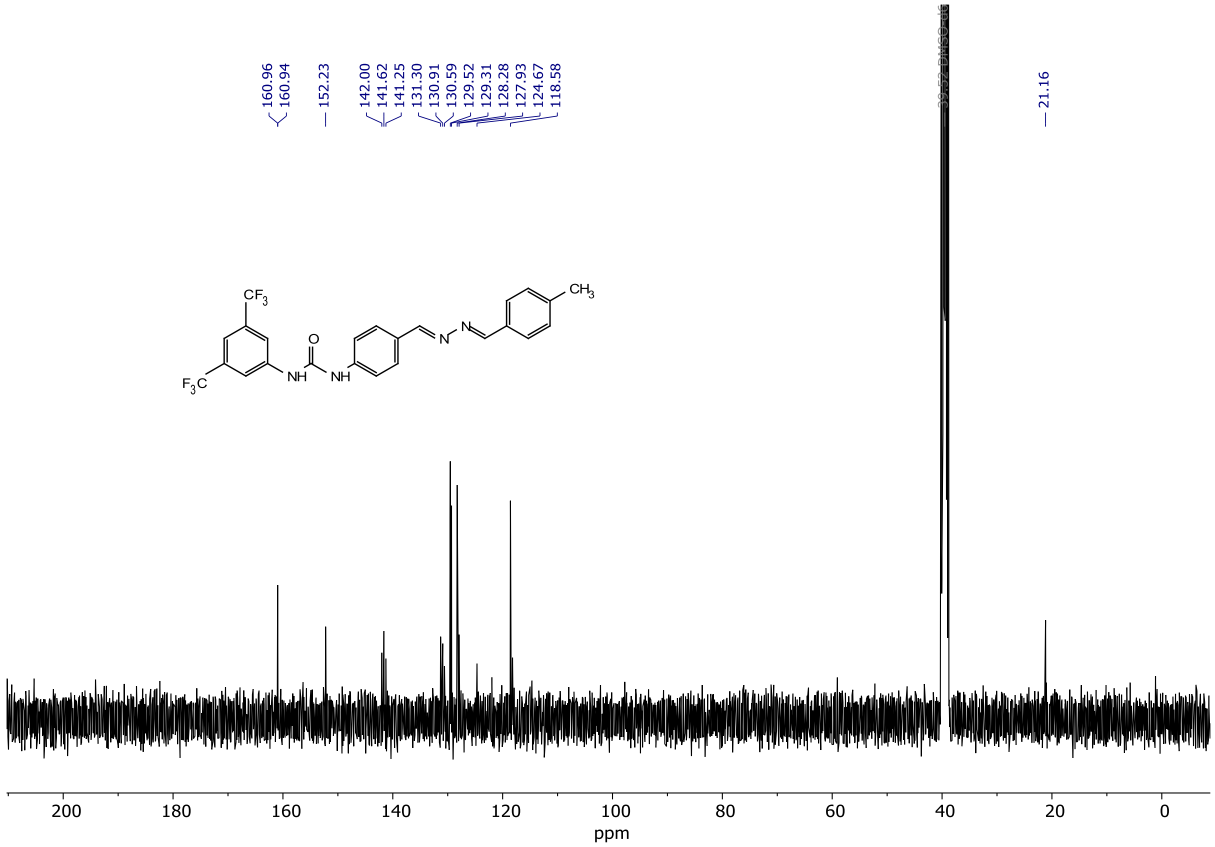

Figure S8. $^{13}C\{^{1}H\}$ NMR spectrum of **TA** (DMSO-d_6, 100 MHz, 298 K).

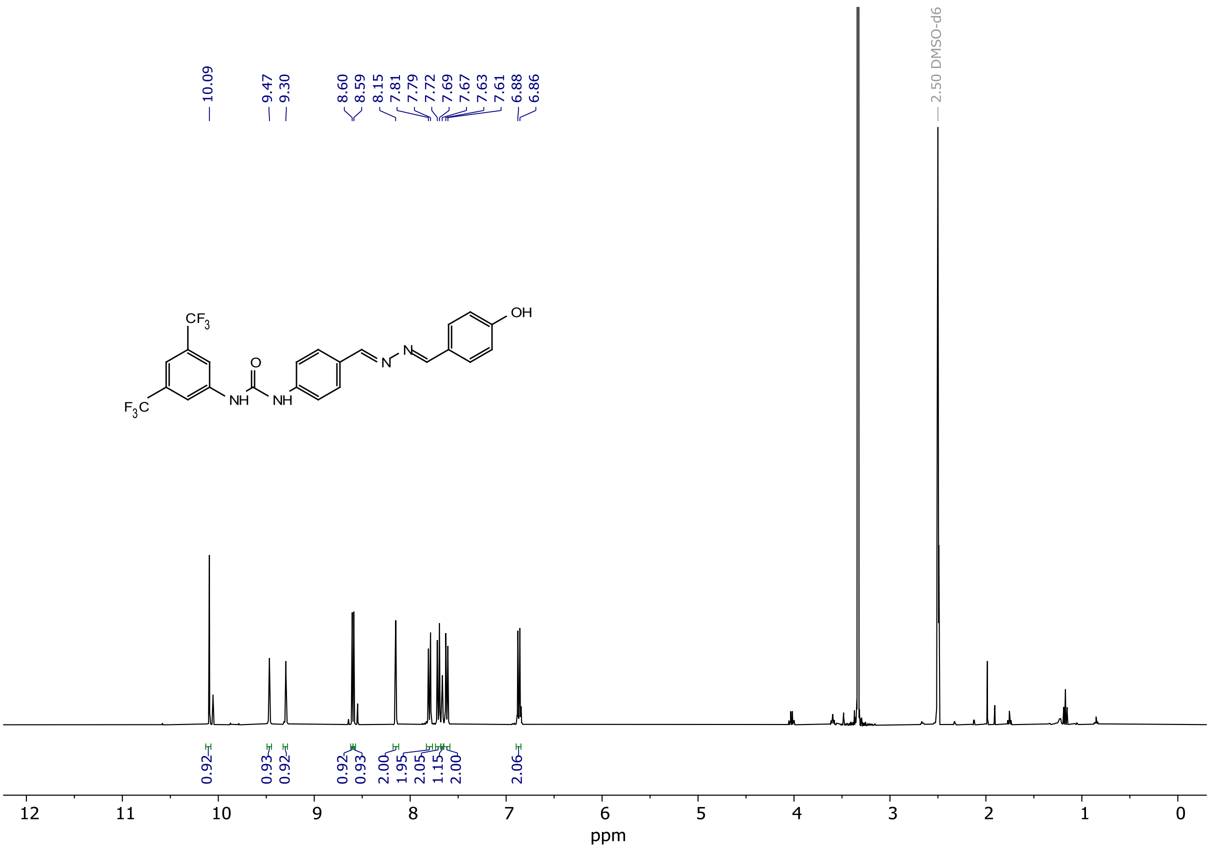

Figure S9. ^{1}H NMR spectrum of **TB** (DMSO-d_6, 400 MHz, 298 K).

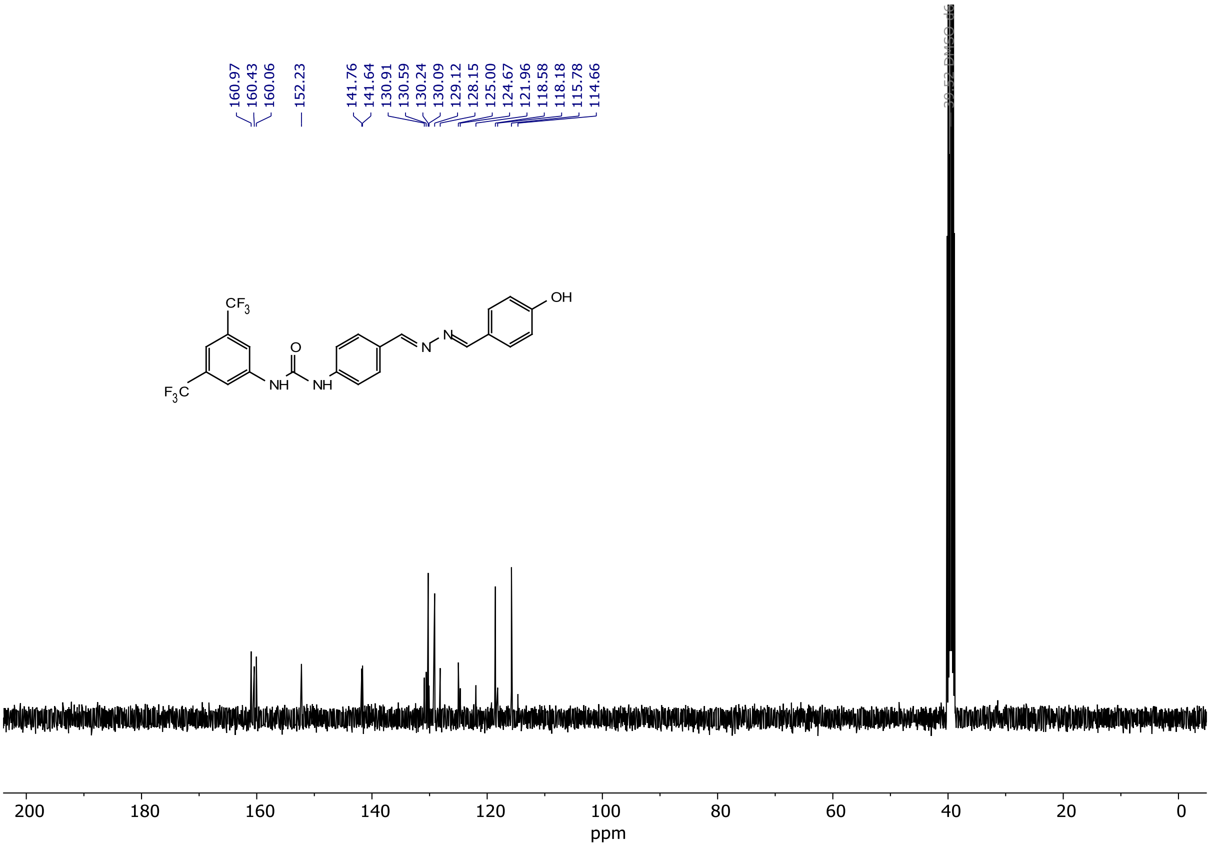

Figure S10. $^{13}C\{^{1}H\}$ NMR spectrum of **TB** (DMSO-d_6, 100 MHz, 298 K).

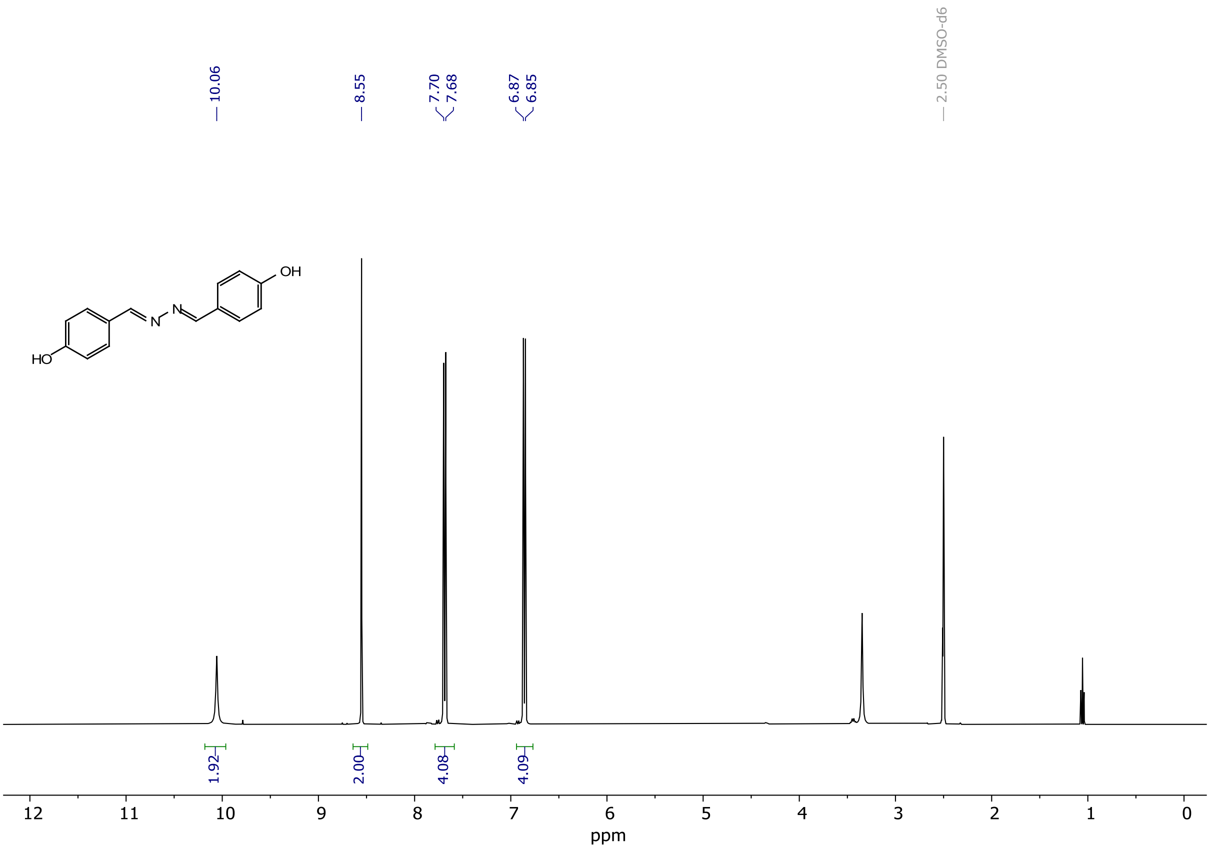

Figure S11. ^{1}H NMR spectrum of **BB** (DMSO-d$_6$, 400 MHz, 298 K).

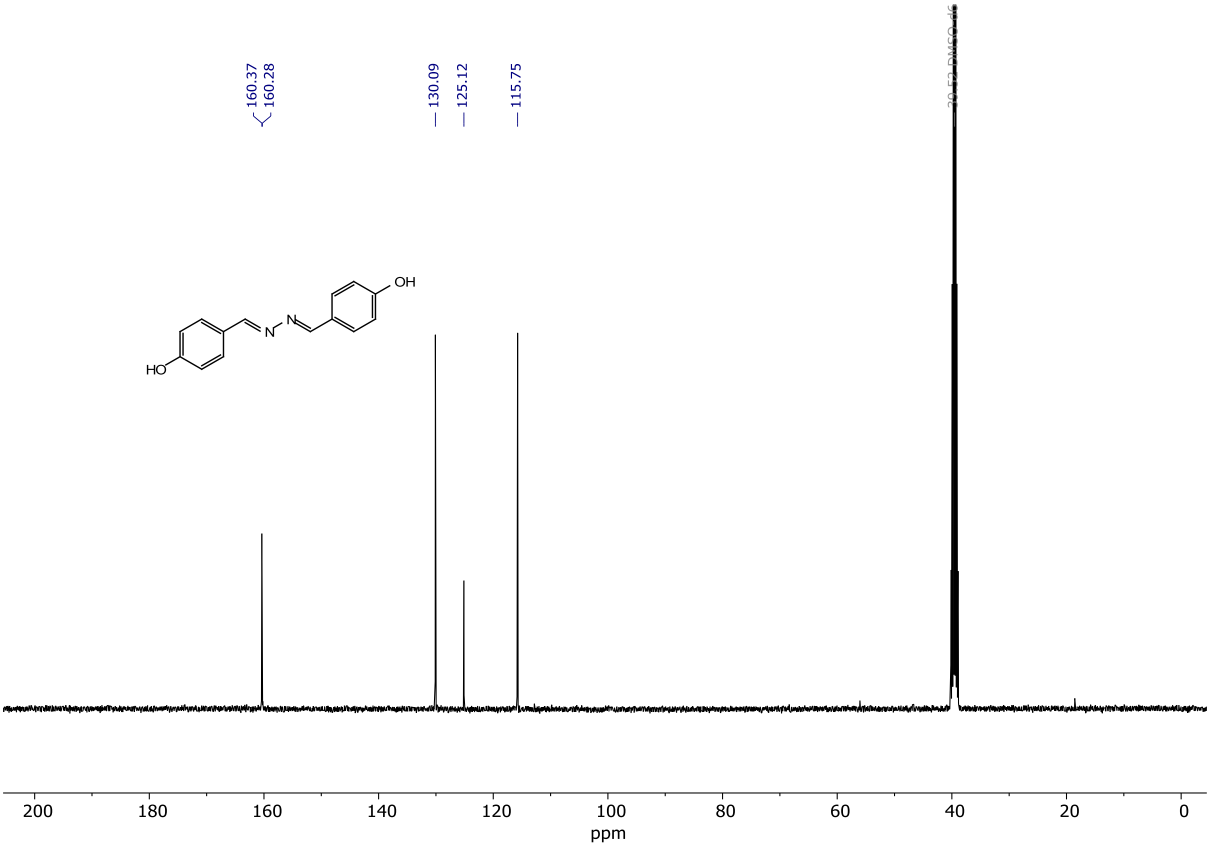

Figure S12. $^{13}C\{^{1}H\}$ NMR spectrum of **BB** (DMSO-d$_6$, 100 MHz, 298 K).

5. NMR experiments on azine metathesis

Symmetrical azines solutions (250 µL, 10 mM in DMSO-d_6) were mixed equimolarly in NMR tube, resulting in a total volume of 500 µL. For the acidified sample, trifluoroacetic acid (TFA) was added (1 equiv., 10 µL, 250 mM in DMSO-d_6).
For symmetrical azines **AA** and **TT**, the two azine protons give a single singlet signal, while for the asymmetrical azine **TA**, signals from both azine protons are observed separately, leading to 4 signals in the spectrum of the equilibrated mixture (Figure S13). The mixture reached an almost statistical distribution of products with a 1 **AA** : 2 **TA** : 1 **TT** ratio (30:51:19) based on integration via peak fitting. The experiment was repeated with azines **EE** and **TT**, giving similar results, but with azine signals that are further apart and thus easier to distinguish (Figure S14).

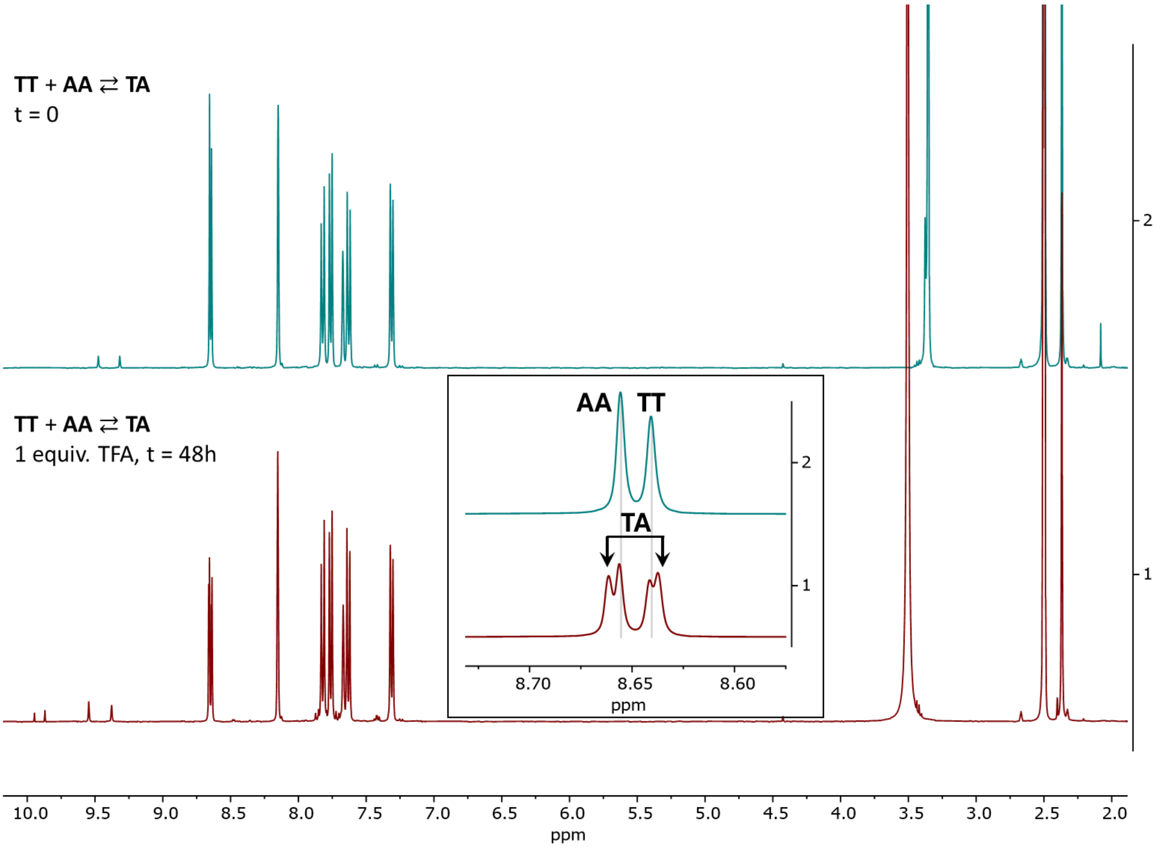

Figure S13. ^{1}H NMR spectra (DMSO-d_6, 400 MHz, 298 K) of the mixtures of azines **AA** and **TT** without acid and upon equilibration (48h after addition of TFA).

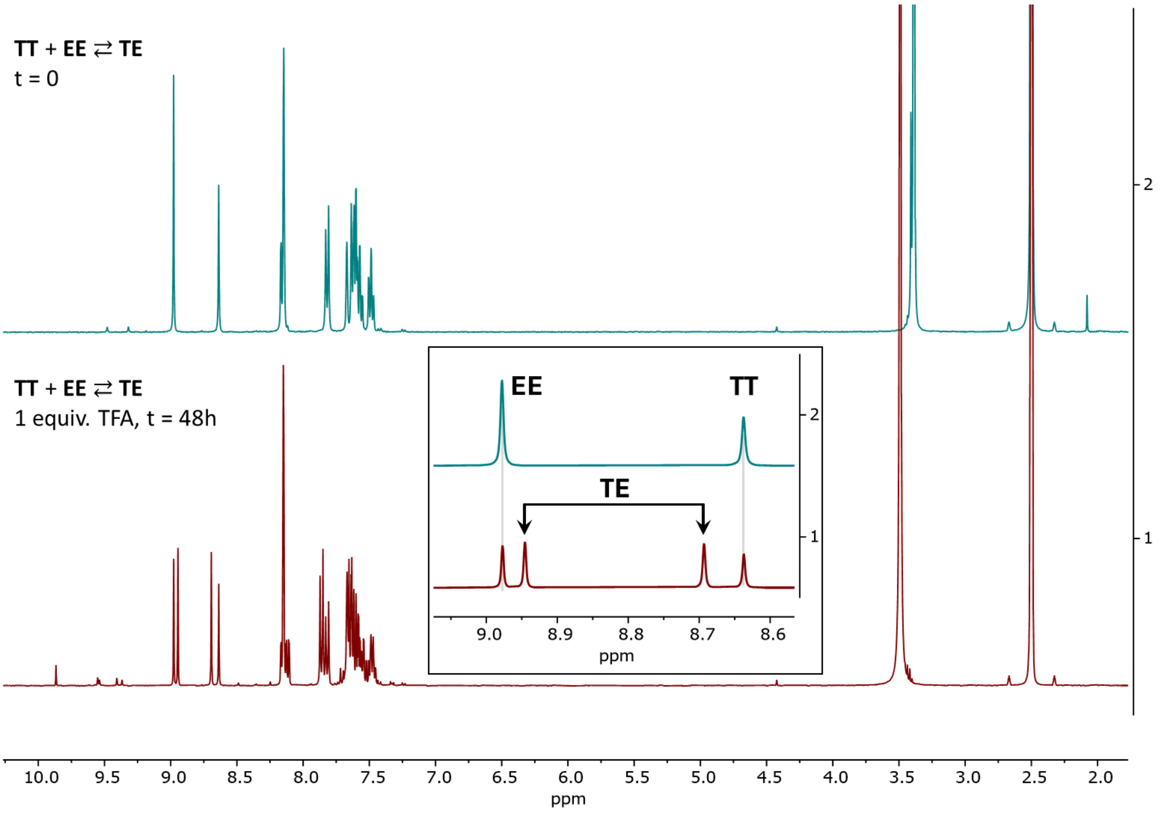

Figure S14. ^{1}H NMR spectra (DMSO-d_6, 400 MHz, 298 K) of the mixtures of azines **EE** and **TT** without acid and upon equilibration (48h after addition of TFA).

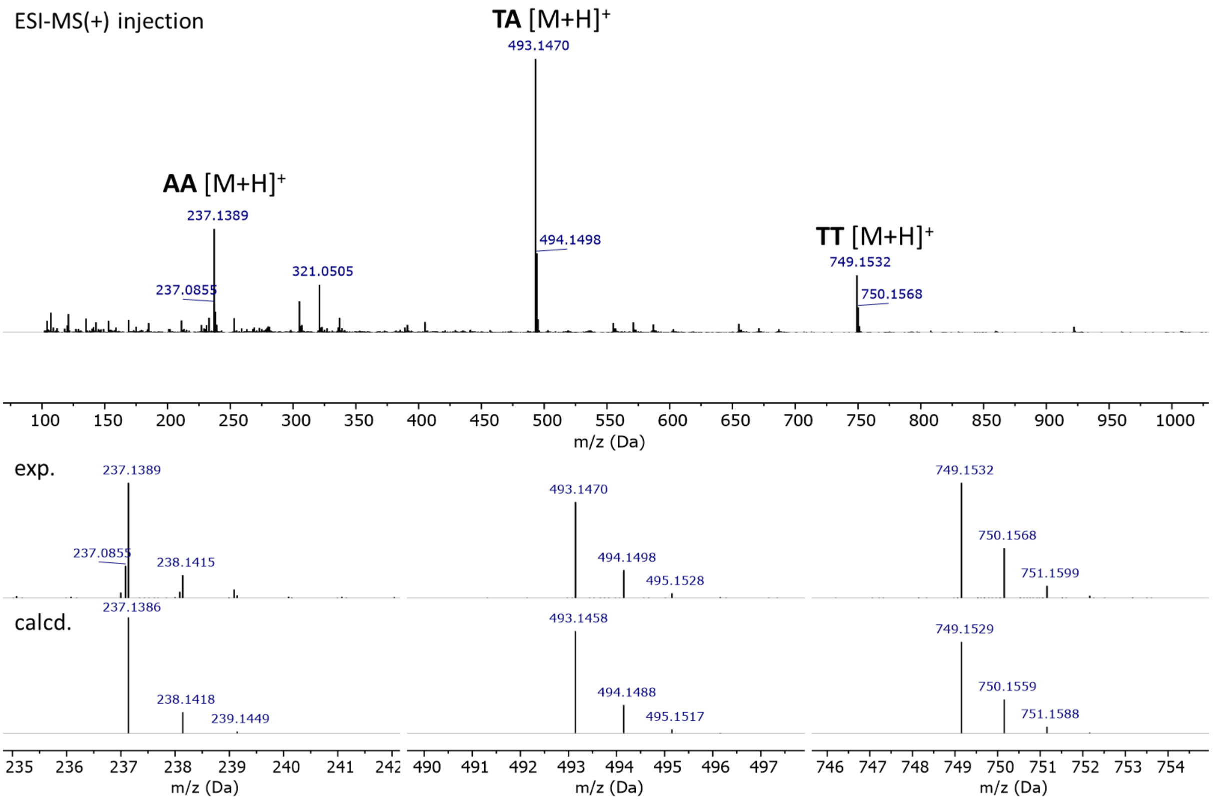

Figure S15. HRMS data on the equilibrated mixture of the **TT** + **AA** ⇄ **TA** azines mixture (with TFA, in DMSO-d_6) from the experiment in Figure S13.

6. Transport studies with the lucigenin assay

Liposome preparation

POPC (1-Palmitoyl-2-oleoylphosphatidylcholine) and **cholesterol** solutions (15-30 mM) in deacidified chloroform were combined in a 5 mL round bottom flask. The volumes of the aliquots were calculated from the concentrations of the lipid solutions to obtain a mixture containing 7 µmol of POPC and 3 µmol of cholesterol. To preincorporate a compound into the liposomal membrane, a stock solution of the compound in organic solvent was added to the mixture (**TA** and **TB** in MeCN 2.0 mM, and **TT** in THF 2.0 mM). The added volume of the stock solution was calculated to obtain the 1.0 mol% compound: lipid ratio. The solvents were evaporated under a gentle flow of nitrogen and the resulting lipid film was dried under a high vacuum for at least 1h. After that, the lipid film was hydrated with 500 µL of an aqueous solution of lucigenin dye (N,N'-dimethyl-9,9'-biacridinium dinitrate, 0.8 mM) or SPBA dye (bis(3-sulfopropyl)-9,9'-biacridine, 0.8 mM)[3] in a solution of the buffer (225 mM $NaNO_3$, 5 mM HEPES for pH 7.0; or 225 mM $NaNO_3$, 5 mM MES for pH 5.0). The resulting mixture was sonicated for 30 s and stirred for 1h to give heterogeneous vesicles. Multilamellar vesicles were disrupted by 10 freeze-thaw cycles using liquid nitrogen. Next, the mixture was diluted to 1 mL (by adding 0.5 mL of buffer solution) and extruded 29 times through a polycarbonate membrane with 200 nm pores in a mini-extruder (Avestin LiposoFast-Basic). The external dye was then removed by passing the liposomes through a pre-packed size exclusion column (containing 8.3 mL Sephadex G-25 medium) and eluted with buffer solution. The collected LUVs were further diluted with buffer solution to the total volume of 25 mL (0.4 mM of lipids) and used for transport measurements right after preparation.

Anion transport measurements

The transport studies have been recorded on a Horiba Fluoromax-4 spectrometer. The spectrometer was to be turned on at least 30 minutes before measurements to warm up the lamp. The sample chamber is thermostated at 25°C. For each run, the 3.00 mL of freshly prepared liposome solution was placed in a quartz cuvette with a small stirrer bar, and the cuvette was placed in the sample chamber. The transporter was added to the liposomes as a solution in an organic solvent (MeCN or MeOH) by a pipette. The sample temperature was stabilized by stirring at 25°C for 3 minutes (these conditions were maintained throughout the transport experiment), and when equilibrium was reached, transport measurements were started. The transport was then recorded by observing fluorescence intensity over time. Fluorescence was monitored at 505 nm (15 minutes, 0.2 s interval, 3 nm slits) with excitation at 430 nm (3 nm slits). For transport measurements, 75 µL of NaCl (1 M, in 225 mM $NaNO_3$) was added to the liposomes 30 s after the start of fluorescence recording to reach a Cl^- concentration gradient of 25 mM. 10 minutes after Cl^- addition the liposomes were lysed by the addition of 50 µl of Triton X-100 (5% wt/wt. in water).

Each experiment was performed at least twice and the data were normalized based on the starting fluorescence levels (F/F_0). These normalized data are provided in the ESI, while the figures in the main text show the further averaged data after removal of the initial plateau (before addition of NaCl) and drop (due to quenching of traces of non-encapsulated probe) as described previously.[3]

[3] M. Chvojka, A. Singh, A. Cataldo, A. Torres-Huerta, M. Konopka, V. Šindelář and H. Valkenier, The Lucigenin Assay: Measuring Anion Transport in Lipid Vesicles, *Analysis & Sensing*, 2023, **4**, e202300044.

Results of the anion transport measurements

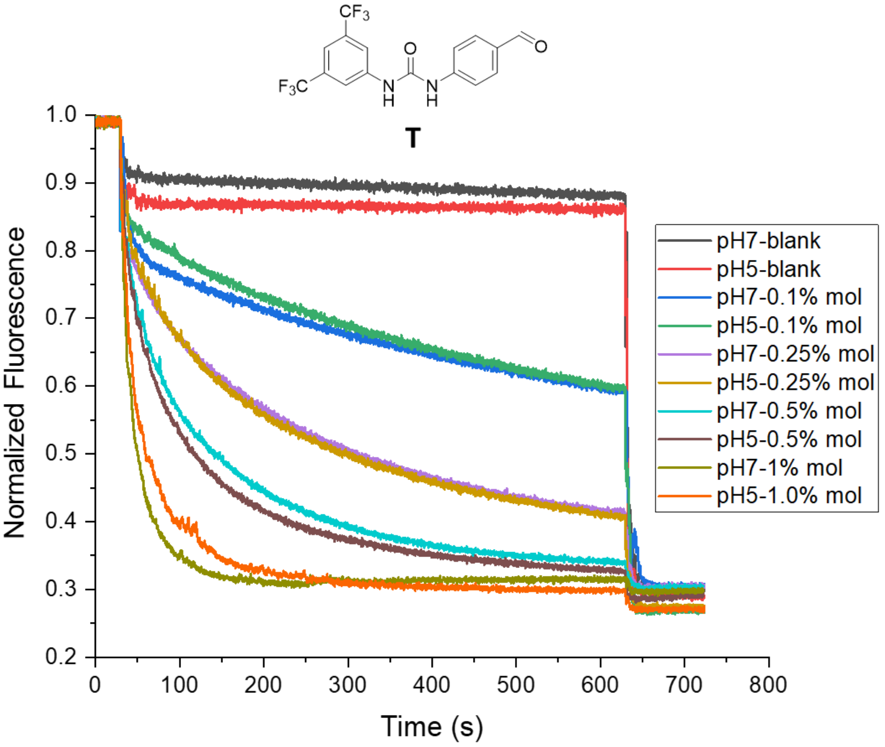

Figure S16. Comparison of transporter **T** transport activity in the lucigenin assay in different pH conditions (pH 5.0 – 5 mM MES, pH 7.0 – 5 mM HEPES).

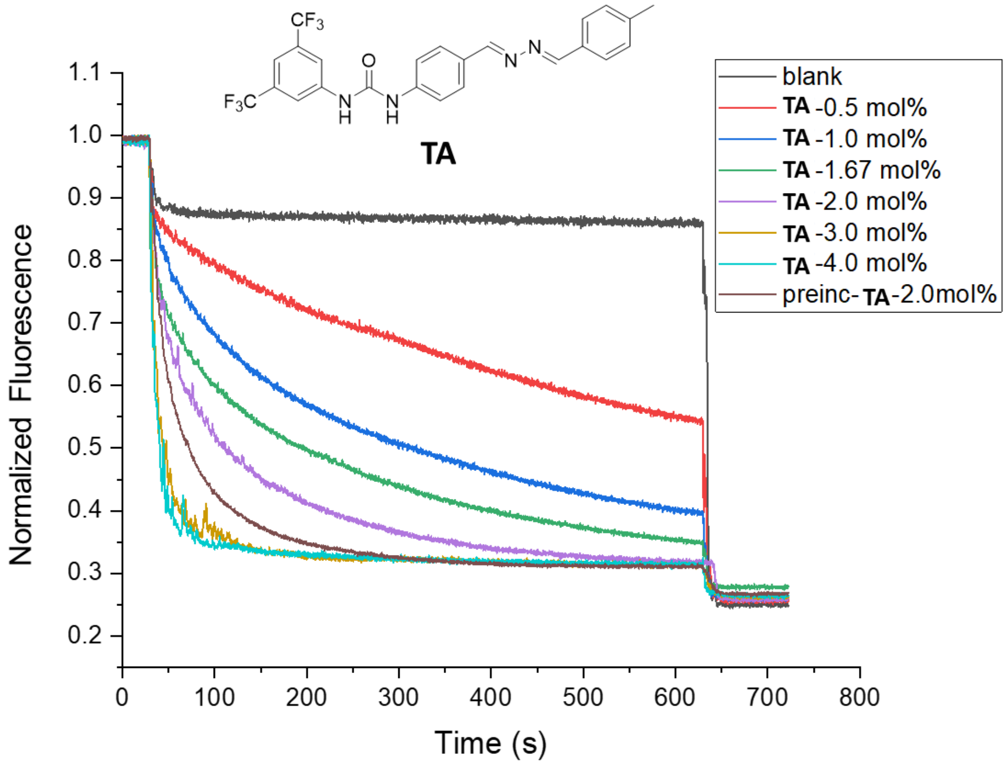

Figure S17. Transporter **TA** transport activity in the lucigenin assay at pH 5.

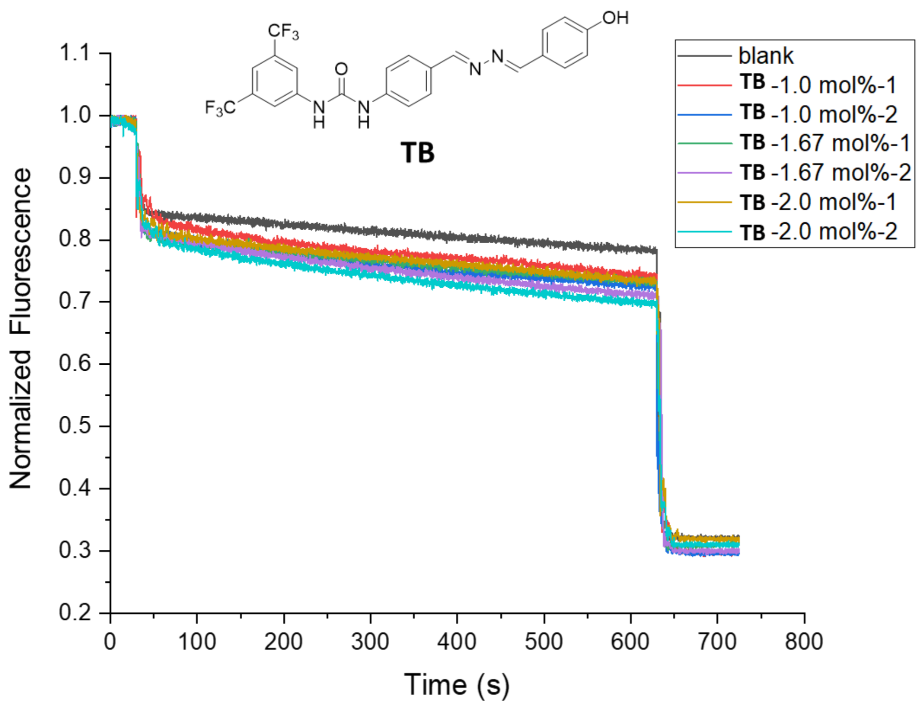

Figure S18. Transporter **TB** transport activity in the lucigenin assay – post-inserted at pH 5.

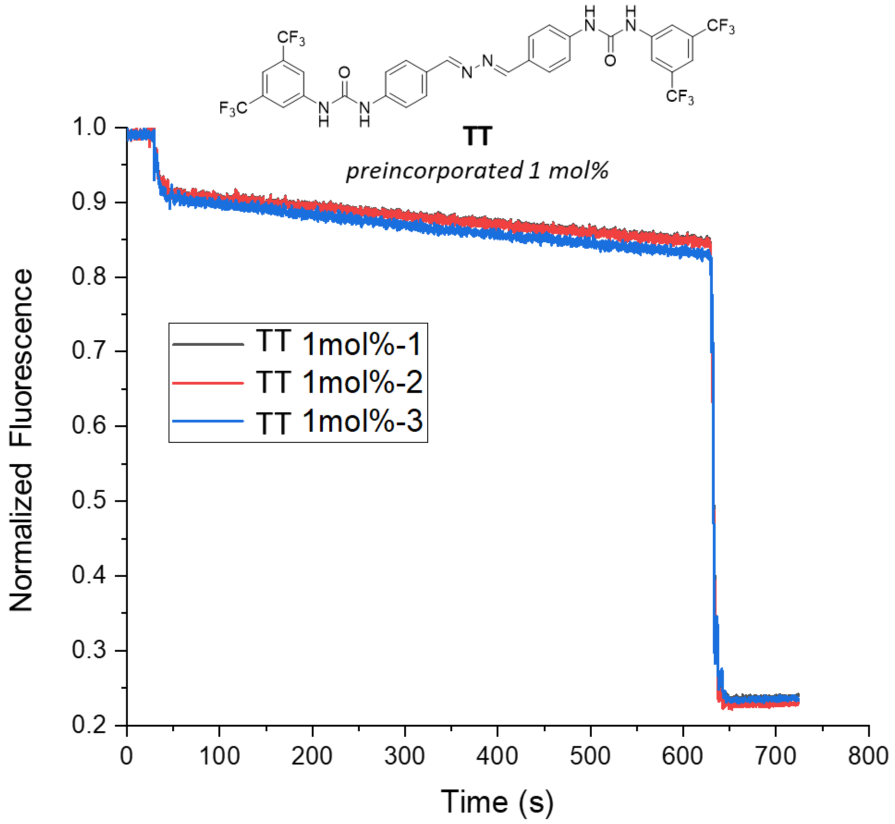

Figure S19. Transporter **TT** transport activity in the lucigenin assay – preincorporated at pH 5.

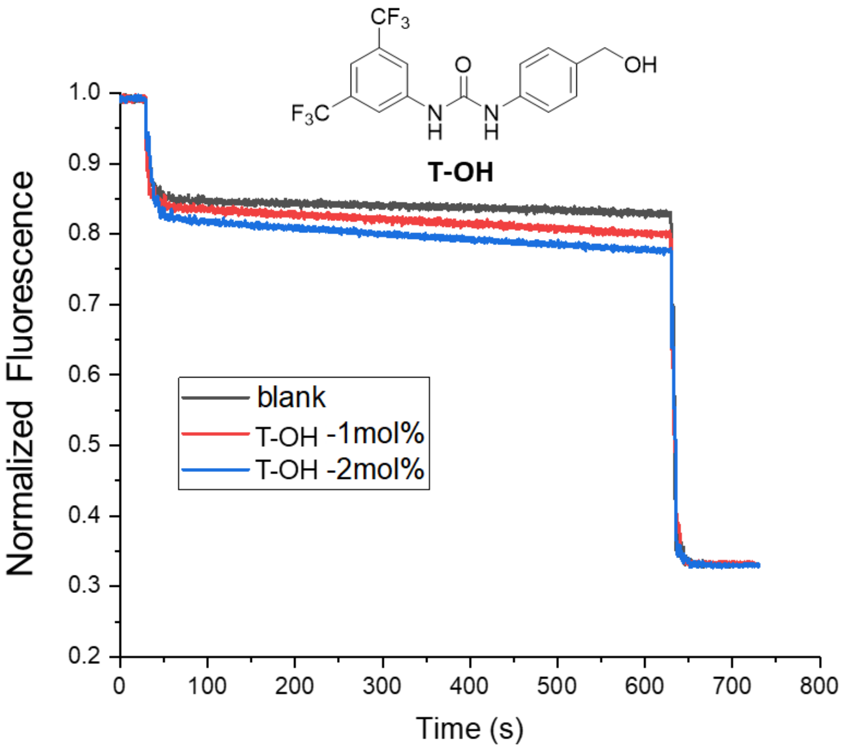

Figure S20. Transporter **T-OH** transport activity in the lucigenin assay – post-inserted at pH 5.

Experiment conditions:
LUCIGENIN 0.8 mM (internal).
Buffer: 225 $NaNO_3$, 5 mM MES, **pH 5.1**

Azines **AA-FF -** 5 mol% (MeCN solution, 10 μL) were post-inserted 2 h before run.
1.0 M NaCl – 75 μL pulse - added 30 s after run.
Triton X-100 (5% wt.) – 50 μL pulse added 630 s after run.

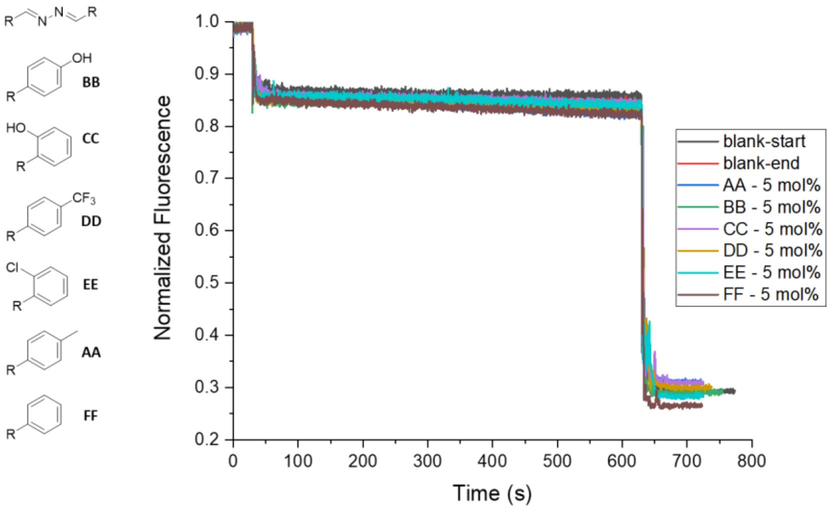

Figure S21. Azines **AA-FF** transport activity in the lucigenin assay at pH 5 after 2h.

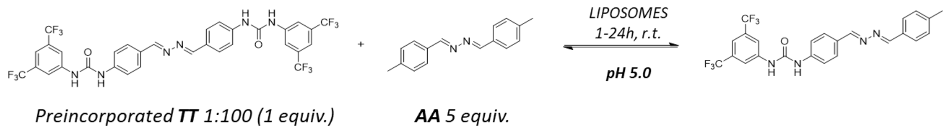

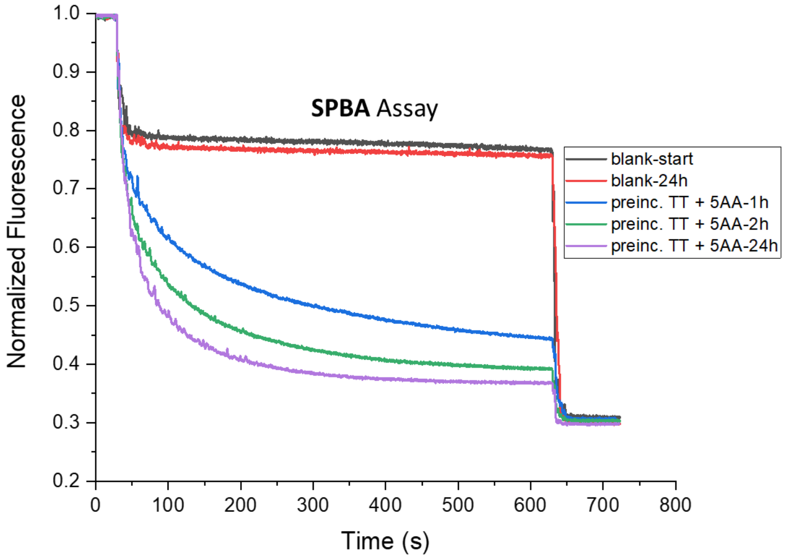

Experiment conditions:
SPBA 0.8 mM (internal).
Buffer: 225 $NaNO_3$, 5 mM MES, **pH 5.1**
Azine **TT** was preincorporated in 1 mol% (1:100 ratio).
AA - 5 mol% (MeCN solution, 10uL) was post-inserted 2-24 h before run.
1.0 M NaCl – 75 µL pulse - added 30 s after run.
Triton X-100 (5% wt.) – 50 µL pulse added 630 s after run.

Figure S22. Long scale time-dependent (0 – 24h) observation of the metathesis progress of preincorporated **TT** (1.0 mol%) with post-inserted **AA** (5.0 equivs.) using SPBA assay at pH 5.0.

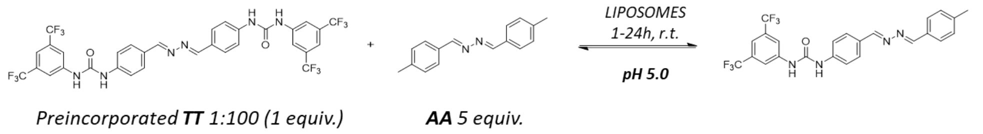

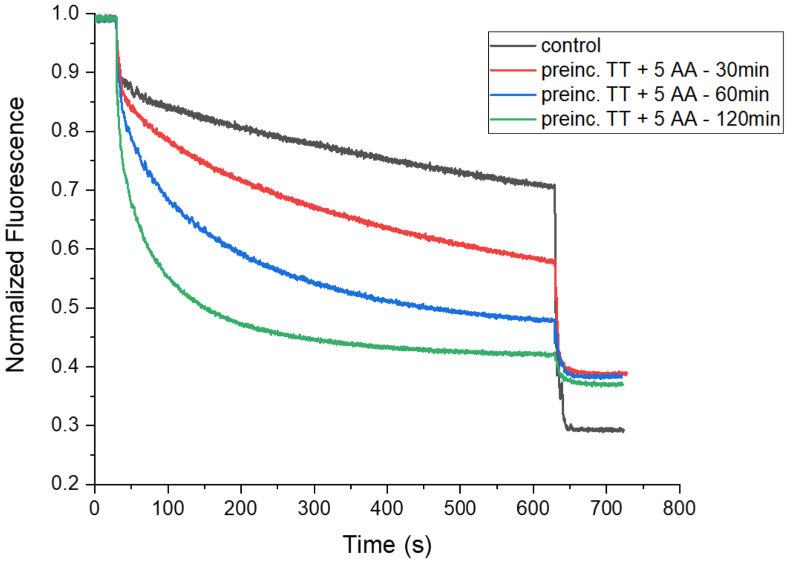

Experiment conditions:
LUCIGENIN 0.8 mM (internal).
Buffer: 225 $NaNO_3$, 5 mM MES, **pH 5.1**
Azine **TT** was preincorporated in 1 mol% (1:100 ratio).
AA - 5 mol% (MeCN solution, 10uL) were post-inserted 0.5-2 h before run.
1.0 M NaCl – 75 µL pulse - added 30 s after run.
Triton X-100 (5% wt.) – 50 µL pulse added 630 s after run.

Figure S23. Time-dependent (0.5 – 2h) observation of the azine metathesis progress of preincorporated **TT** (1.0 mol%) with post-inserted **AA** (5.0 equivs.) using lucigenin assay at pH 5.0.

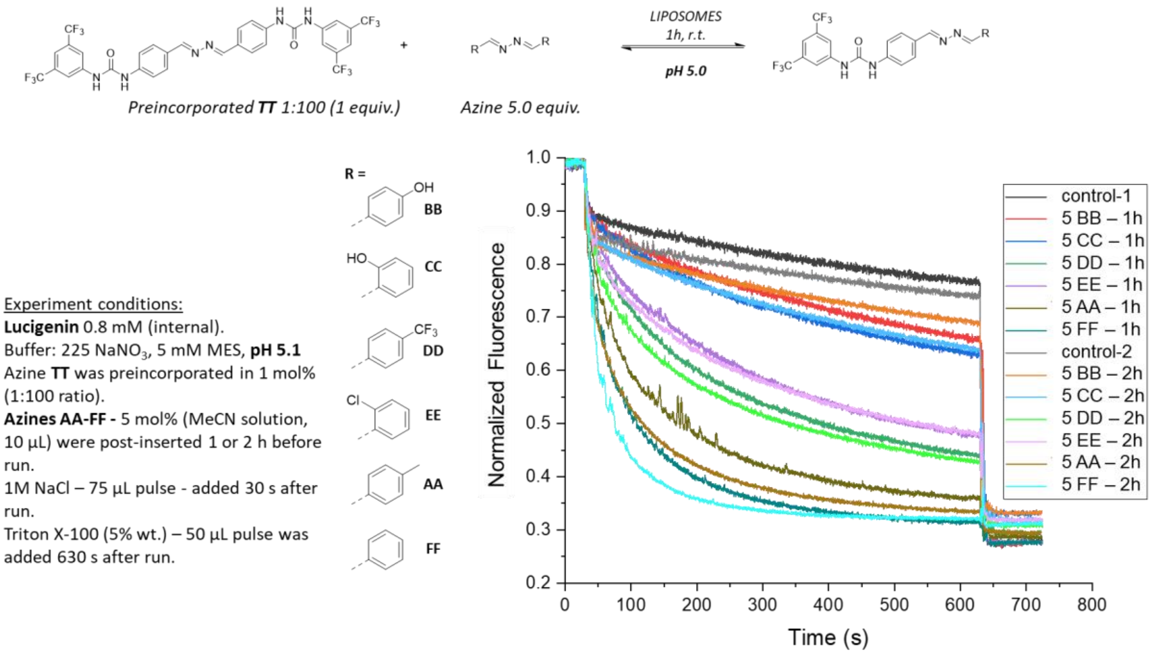

Figure S24. Liposomes with preincorporated **TT** (1.0 mol%) at pH 5.0 (5.0 mM MES) treated with various symmetrical azines (post-inserted 5 equiv., r.t., 1h and 2h).

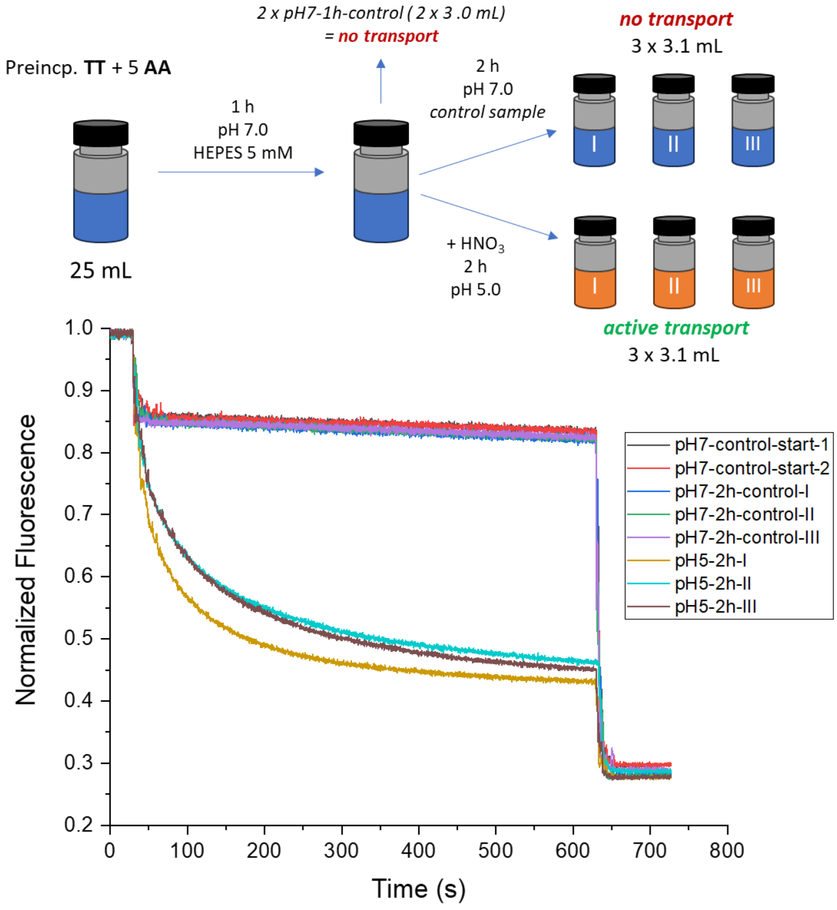

Figure S25. Controlling the system by pH. Preincorporated **TT** (1.0 mol%) and azine **AA** (5 equiv.) starting from pH 7.0 (1h) and switching on with pH 5.0 (1h).

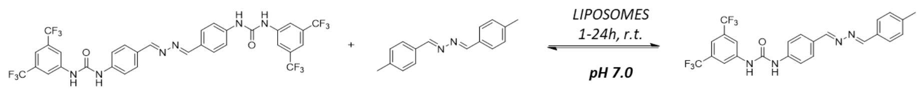

*Preincorporated **TT** 1:100 (1 equiv.)* ***AA** 5 equiv.*

<u>Experiment conditions:</u>
SPBA 0.8 mM (internal).
Buffer: 225 $NaNO_3$, 5 mM HEPES, **pH 7.0**
Azine **TT** was preincorporated in 1 mol% (1:100 ratio).
AA - 5 mol% (MeCN solution, 10 µL) was post-inserted 2-24 h before run.

1M NaCl – 75 µL pulse - added 30 s after run.
Triton X-100 (5% wt.) – 50 µL pulse added 630 s after run.

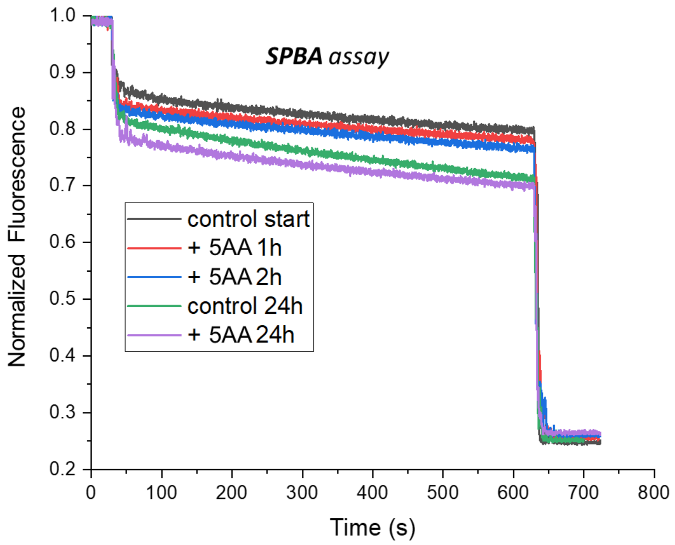

Figure S26. Long scale time-dependent (0 – 24 h) observation of the metathesis progress of preincorporated **TT** (1.0 mol%) with post-inserted **AA** (5.0 equivs.) using the SPBA assay at pH 7.0.

7. Dynamic products distribution

The statistical distribution of the dynamic products that are formed during the azine metathesis reaction in the membrane has been calculated.

Table S1. The reaction between preincorporated **TT** (1 mol%) and post-inserted **AA** (5 mol%) – 6 mol% of azines in total.

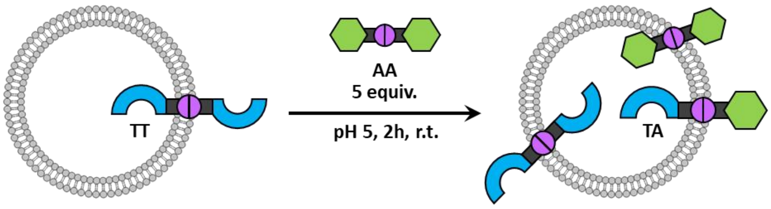

Product	Product fraction	mol%
**TA**	10/36	1.67
**TT**	1/36	0.17
**AA**	25/36	4.17

Table S2. The reaction between preincorporated **TB** (1 mol%) and post-inserted **AA** (5 mol%) – 6 mol% of azines in total.

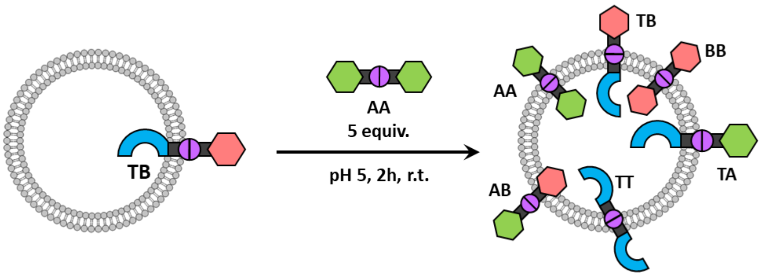

Product	Product fraction	mol%
**TB**	2/144	0.08
**TA**	20/144	0.83
**TT**	1/144	0.04
**AA**	100/144	4.17
**BB**	1/144	0.04
**BA**	20/144	0.83

8. Membrane fluidity impact on azines metathesis

DPPC (dipalmitoylphosphatidylcholine, 12.2 mg, 16.6 µmol) was dissolved in deacidified chloroform. In the next step, 1.0 mol% of **TT** was preincorporated from THF solution (33 µL, 5 mM). Solvents were then removed under a flow of nitrogen, and the lipid film was dried under a high vacuum for 1h.

The dried **TT+DPPC** lipid film was hydrated with lucigenin solution (0.8 mM, 0.5 mL, 225 mM $NaNO_3$, 5 mM MES, pH 5). The mixture was then sonicated for 3 min. and stirred at 45°C for 1h. Then the mixture was freeze-thawed 10 times with a water bath at 50°C. Hydrated lipids (500 µL + buffer 500 µL) were then extruded 29 times through a polycarbonate membrane with 200 nm pores using a preheated extruder at 45°C. Then the LUVs were isolated from external lucigenin with a size exclusion column and diluted to a concentration of 0.4 mM with buffer 41 mL (225 mM $NaNO_3$, 5 mM MES, pH 5). Compound **AA** (5 mol%) was added to these DPPC LUVs and transport were performed using the lucigenin assay as described in Section 6 at both 25 and 45°C.

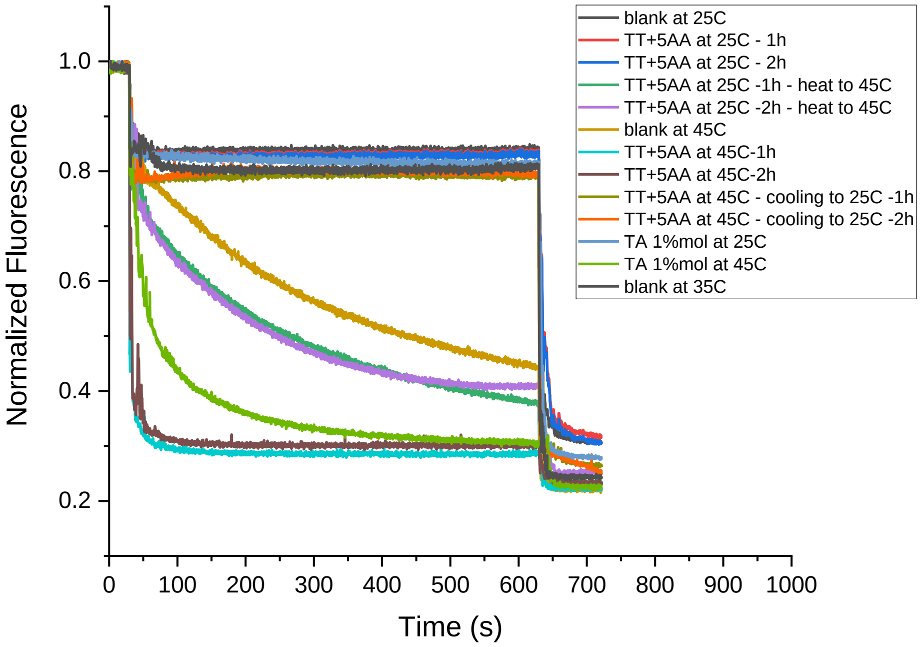

Figure S27. Membrane fluidity impact on azine metathesis and transport. Transport curves registered at 25°C (DPPC in the solid ordered phase) and 45°C (DPPC in the liquid disordered phase).

Table S3. Results of DPPC-based LUVs experiments.

**Experiment**	**Transport Temp. (°C)**	**Transport?**
Blank at 25°C	25	No
**TA** 1 mol% post-inserted at 25°C	25	No
**TT** + 5 **AA** at 25°C, equilibrated for 1h	25	No
**TT** + 5 **AA** at 25°C, equilibrated for 2h	25	No
**TT** + 5 **AA** at 45°C – 1h then cooled down to 25°C	25	No
**TT** + 5 **AA** at 45°C – 2h then cooled down to 25°C	25	No
Blank at 35°C	35	No
Blank at 45°C	45	Leakage
**TA** 1 mol% post-inserted at 45°C	45	Good
**TT** + 5 **AA** at 25°C – 1h then heated up to 45°C	45	Moderate
**TT** + 5 **AA** at 25°C – 2h then heated up to 45°C	45	Moderate
**TT** + 5 **AA** at 45°C, equilibrated for 1h	45	Very good
**TT** + 5 **AA** at 45°C, equilibrated for 2h	45	Very good

9. Study of the incorporation of TT in the liposomal membrane

Compound **TT** is not soluble in water, as demonstrated by the absence of any ^{1}H NMR signals in the spectrum of Figure S28a in D_2O and the solid that is clearly visible in the corresponding NMR tube (left in the photo).

POPC (1-Palmitoyl-2-oleoylphosphatidylcholine, 700 µL, 23 mM) solution in deacidified chloroform was placed in a 5 mL round bottom flask. Next **TT** was preincorporated in 5 mol% from THF solution (160 µL, 5 mM). Solvents were then removed under a gentle flow of nitrogen, and the resulting lipid film was dried under a high vacuum for 1h.
The dried **TT+POPC** lipid film was hydrated with a buffer of 800 µL (225 mM $NaNO_3$ in D_2O, acidified with 3 µL of 1 M HNO_3). The mixture was then sonicated for 1 minute and stirred at r.t. for 1h. Hydrated lipids (800 µL) were then extruded 29 times through 200 nm pores polycarbonate membrane and 21 times through 30 nm pores membrane to obtain small unilamellar vesicles (SUVs). The extrusions were performed an odd number of times, to ensure the effective filtration of the liposome suspension.

As the ^{1}H NMR signals of the SUVs with **TT** were too broad for analysis, these liposomes (100 µL SUVs) were lysed with a mixture of 100 µL $CDCl_3$, 450 µL CD_3OH, and 20 µL D_2O with 15 mM TMSPA-Na.[4] The ^{1}H NMR spectra of the resulting solution showed clear signals of compound **TT** (Figure 28b), of which integration revealed a concentration of ~0.11 mM **TT** and a ratio of ~4 mol% **TT** compared to POPC. These values are close to the maximum theoretical concentration of **TT** (0.15 mM, assuming no losses during sample preparation), implying that **TT** was effectively incorporated in the membrane of the liposomes.

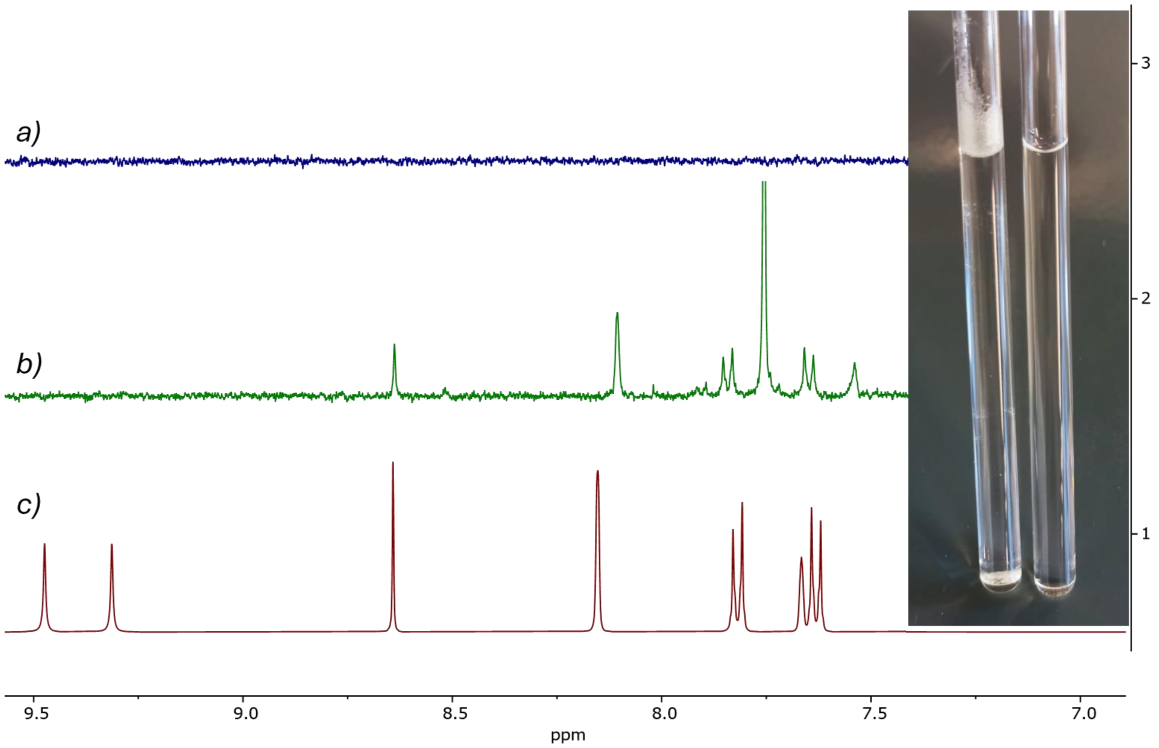

Figure S28. Comparison of ^{1}H NMR spectra (400 MHz, 298 K): a) **TT** in D_2O – insoluble, b) lysed **TT+POPC** liposomes mixture in D_2O + $CDCl_3$ + CD_3OH with clear **TT** signals, c) **TT** in DMSO-d_6 for comparison. Inset: Photo with the left tube referring to spectrum a) – undissolved and hydrophobic solid of **TT**, and the right tube referring to spectrum b) – clear solution, where **TT** is dissolved.

[4] R. Hein, C. B. Uzundal, A. Hennig, *Org. Biomol. Chem.* **2016**, *14*, 2182–2185.
